# Mendelian randomization analysis suggests no causal influence of gastroesophageal reflux disease on the susceptibility and prognosis of idiopathic pulmonary fibrosis

**DOI:** 10.1186/s12890-023-02788-8

**Published:** 2023-12-21

**Authors:** Di Sun, Qiao Ye

**Affiliations:** Department of Occupational Medicine and Toxicology, Clinical Center for Interstitial Lung Diseases, Beijing Institute of Respiratory Medicine, Beijing Chao-Yang Hospital, Capital Medical University, Beijing, 100020 China

**Keywords:** Idiopathic pulmonary fibrosis, Gastroesophageal reflux disease, Mendelian randomization

## Abstract

**Background:**

The relationship between gastroesophageal reflux disease (GERD) and the susceptibility as well as the prognosis of idiopathic pulmonary fibrosis (IPF) has been previously suggested, with the potential confounding factor of smoking not adequately addressed. In light of this, we conducted a Mendelian randomization (MR) study to investigate the causal effects of GERD on the susceptibility and prognosis of IPF while excluding smoking.

**Methods:**

We chose GERD as the exposure variable and employed genome-wide association data to examine its association with susceptibility, forced vital capacity (FVC), diffusing capacity of the lung for carbon monoxide (DLco), and transplant-free survival (TFS) in patients with IPF as the outcome variables. MR analyses were performed using the inverse variance weighted (IVW) method, and sensitivity analyses were conducted using the MR-PRESSO outlier test, Cochran’s Q test, MR-Egger intercept test, and leave-one-out sensitivity analysis. Additionally, to mitigate the potential effects of smoking on our MR estimates, we conducted a multivariable MR (MVMR) analysis by adjusting for smoking.

**Results:**

The univariable MR analysis demonstrated no causal effect of GERD on FVC (*β*_IVW_ = 26.63, SE = 48.23, *P* = 0.581), DLco (*β*_*IVW*_ = 0.12, SE = 0.12, *P* = 0.319), and TFS (HR_*IVW*_ = 0.87, 95% CI = 0.56 to 1.35, *P* = 0.533) in patients with IPF. Furthermore, sensitivity analysis revealed no evidence of heterogeneity, horizontal pleiotropy, or outlier single nucleotide polymorphisms. The MVMR analysis showed no causal effect of GERD on susceptibility to IPF after adjusting for smoking (OR_*IVW*_ = 1.30, 95% CI = 0.93 to 1.68, *P* = 0.071). These findings were consistent in the replication cohort.

**Conclusions:**

The link between GERD and its potential impact on susceptibility to IPF may not be of a direct causal nature and could be influenced by factors such as smoking. Our findings did not reveal any evidence of a causal relationship between GERD and the FVC, DLco, and TFS of patients with IPF.

**Supplementary Information:**

The online version contains supplementary material available at 10.1186/s12890-023-02788-8.

## Background

Idiopathic pulmonary fibrosis (IPF) is a severe and progressive fibrotic lung disease [1]. Patients with IPF have a very poor prognosis, with a median survival of 3–5 years after diagnosis [2, 3], and the survival rate is only 66% at 3 years after lung transplantation [4]. The forced vital capacity (FVC), diffusing capacity of the lung for carbon monoxide (DLco), and transplant-free survival (TFS) were considered to be the key outcomes for assessing the prognosis of IPF [5]. Evidence suggests that epithelial damage and abnormal wound repair contribute to the pathogenesis of IPF, and environmental exposure may be involved in this process, especially in patients with gastroesophageal reflux disease (GERD) [1].

GERD encompasses a constellation of distressing symptoms and complications that arise due to the reflux of stomach contents into the esophagus [6]. Although some studies have suggested that GERD-associated microaspiration may initiate or promote fibrosis and contribute to the disease progression of IPF [7, 8], empirical acid suppression treatment did not slow the progression of IPF [9, 10].

Smoking has been demonstrated to have a causal relationship with an increased susceptibility to both IPF and GERD [11, 12]. Reynolds et al. [13] found that GERD increased the risk of IPF using a bidirectional two-sample Mendelian randomization (MR) study, but they did not account for smoking as a confounding factor or adjust for it, potentially leading to false positive results. Therefore, when investigating the relationship between IPF and GERD, it is imperative to systematically exclude smoking-related SNPs and utilize a multivariate MR (MVMR) study to effectively adjust for smoking.

MR is a statistical technique employed to explore causal relationships between exposures and disease outcomes by utilizing genetic variants as instrumental variables (IVs) [14]. It utilizes the principles of Mendelian inheritance to emulate the design of a randomized controlled trial, thus providing valuable insights into causality within observational studies [14]. In comparison to conventional observational studies, MR can help mitigate bias resulting from confounding factors by utilizing genetic variants as IVs, as these variants are typically unaffected by confounders [15].

Therefore, the objective of this study is to investigate the causal effect of GERD on the susceptibility of IPF while excluding confounding factors, such as smoking. Additionally, we also explored the causal relationship between GERD and the prognosis of IPF using the MR approach.

## Methods

### Study design

**Fig. 1:**
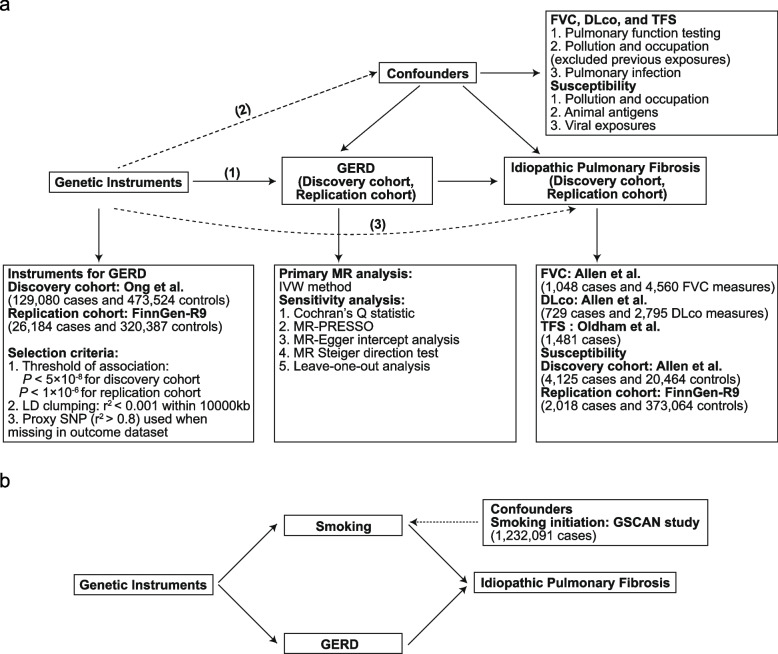
Study design. **a** Univariable MR. **b** Multivariable MR. Abbreviations: MR: Mendelian randomization; IVW: inverse variance weighted; GERD: gastroesophageal reflux disease; FVC: forced vital capacity; DLco: diffusing capacity of the lungs for carbon monoxide; TFS: transplantation-free survival; SNP: single nucleotide polymorphism

The overall research process of this study is illustrated in Fig. 1. First, the causal effect of GERD on susceptibility to IPF was estimated using the univariable MR approach. Second, a univariable MR design was employed to identify the causal effect of GERD on FVC, DLco, and TFS in the patients with IPF. Third, to account for the effects of smoking on the MR estimates, a MVMR analysis was conducted. Finally, the aforementioned results in the replication cohort were further validated.

### Mendelian randomization

This study adheres to the guidelines for Strengthening the Reporting of Mendelian Randomization Studies (STROBE-MR) checklist [16, 17]. The primary analysis employed in this study involved a two-sample MR design. MR approach is based on three fundamental assumptions [18, 19]: (1) the genetic variants exhibit strong associations with the target exposure, (2) the genetic variants are not associated with confounding factors, and (3) the variants do not independently influence the outcome apart from their effect on the exposure.

## Data sources

The genome-wide association studies (GWAS) data for GERD were obtained from Ong et al. (129,080 cases and 473,524 control subjects) [20]. The outcome datasets consisted of GWAS summary statistics for susceptibility (4125 cases and 20,464 control subjects) [21], FVC (1048 cases and 4560 FVC measures) [22], DLco (729 cases and 2795 DLco measures) [22], and TFS (1481 cases, where endpoint events were defined as death or lung transplant) [23] in the patients with IPF (Table 1). For replication, we extracted summary statistics for GERD (26,184 patients and 320,387 control subjects) and susceptibility of IPF (2018 patients and 373,064 control subjects) from the FinnGen biobank (Table 1) [24]. Smoking initiation (1,232,091 patients) was obtained from the GSCAN study (Table 1) [25]. For the FVC analysis the effect size is in terms of change in FVC in ml/year and for the DLco analysis the effect size is in terms of a change of mmol/min/kPa/year. Additionally, we collected the single nucleotide polymorphism (SNP) ID numbers (rs#) for TFS from dbSNP version 151, using version hg19 as the human reference genome [26].

**Table 1:** GWAS summary statistics: source and description

Variables	Data source	PMID	N cases	Sample size	Ethnicity
**GWAS of gastroesophageal reflux disease**					
GERD (discovery cohort)	Ong et al. (2022)	34,187,846	129,080	602,604	European
GERD (replication cohort)	FinnGen-R9	36,653,562	26,184	346,571	European
**GWAS of idiopathic pulmonary fibrosis**					
FVC	Allen et al. (2023)	35,985,358	–	1048	European
DLCO	Allen et al. (2023)	35,985,358	–	729	European
TFS	Oldham et al. (2023)	36,780,644	–	1481	European
Susceptibility (discovery cohort)	Allen et al. (2022)	35,688,625	4125	24,589	European
Susceptibility (replication cohort)	FinnGen-R9	36,653,562	2018	375,082	European
**GWAS of smoking**					
Smoking initiation	Liu M et al. (2019)	30643251	1,232,091	–	European

*Abbreviations*: *GWAS* genome-wide association studies, *GERD* gastroesophageal reflux disease, *FVC* forced vital capacity, *DLco* diffusing capacity of the lungs for carbon monoxide, *TFS* transplantation-free survival

### Instrument selection

The genetic instruments were derived from a large genetic atlas of GERD [20, 24]. SNPs that reached a significance level of *P* < 5 × 10^−8^ (*P* < 1 × 10^−6^ for replication cohort) were clumped for independence based on linkage disequilibrium (r^2^ < 0.001 within 10,000 kb), using the European reference panel from the 1000 Genome Project [27]. In cases where there were limited accessible SNPs for the outcomes, proxy SNPs with a high degree of linkage disequilibrium (r^2^ > 0.8) were employed. The effects of the SNPs on exposures and outcomes were then harmonized to ensure that the beta values were assigned to the same alleles. Outliers were detected using the MR-PRESSO method (defined as those with *P* > 0.05) [28], but no outliers were found in the data. Subsequently, we manually screened and removed SNPs related to confounding factors and outcomes using the PhenoScanner database (*P*-value < 1 × 10^−5^, r^2^ = 0.8, Proxies = EUR, Build = 37) [29, 30]. The results of this screening are presented in Table S1. For the susceptibility to IPF, potential confounders included pollution, occupation, animal antigens, and viral exposures [1, 31]. The remaining SNPs were used to perform the MR study. For FVC, DLco, and TFS in patients with IPF, potential confounders included pulmonary function testing, pollution, occupation, and pulmonary infection (excluded previous exposures) [1, 31].

### Testing instrument strength

To assess the instrument strength for GERD, we employed two parameters: the proportion of variance (R^2^) and the *F*-statistic. The R^2^ was calculated using the formula R^2^ = 2 × EAF × (1–EAF) × *β*^2^ [32], while the *F*-statistic was computed as *F* = *β*^2^ / SE^2^ [33]. The *F*-statistic is considered a measure of instrument strength, and a value greater than 10 indicates a sufficiently strong instrument [34]. All *F*-statistics of the SNPs in our study are ≥30, indicating a robust strength of genetic instruments (Table S2).

### Sensitivity analysis

The primary MR analysis was conducted using the inverse-variance weighted (IVW) method, which provides an unbiased estimate in the absence of horizontal pleiotropy and heterogeneity [35]. Additionally, we performed other methods, including MR-Egger [36], weighted median [37], simple mode [38], and weighted mode under different assumptions [38]. However, the IVW method was preferentially applied when no heterogeneity and horizontal pleiotropy were present. To assess for heterogeneity and horizontal pleiotropy, we performed various tests, including the MR-Egger intercept test [39], Cochran’s Q test [40], and leave-one-out analyses [41]. Lastly, we performed the MR-Steiger directionality test to evaluate the correct direction of causality in the presence of a causal association [42].

### Multivariable Mendelian randomization analysis

Since the effects of GERD on the susceptibility of IPF may also be influenced by smoking [11], a MVMR analysis were conducted. In this analysis, GERD and smoking initiation was used as exposure variables to account for potential confounding by smoking. Two types of MVMR analyses were performed, namely multivariable IVW regression [43] and MVMR-Egger regression [44], as additional sensitivity analyses. In the MVMR approach, all genetic instruments, eliminated duplicate SNPs, and excluded correlated SNPs (r^2^ ≥ 0.001) based on the minimum *P*-value for genetic association with each trait were combined. Subsequently, the associations of the remaining SNPs with both the exposure and outcome variables and fitted multivariable models were extracted.

### Statistical analysis

All statistical analyses were performed using the “TwoSampleMR” package [45], “MRPRESSO” package [28], and “MVMR” package [46] in R (version 4.2.2) with RStudio (version 2022.07.2 Build 576). The threshold for statistical significance was set at *P*-values below 0.05.

## Results

### Effect of GERD on susceptibility to IPF

As in previous studies, we obtained 75 SNPs as IVs to assess the genetic association of GERD with susceptibility to IPF (Tables S2 and S3). The results of the IVW method showed a causal effect of GERD on susceptibility to IPF (odds ratio (OR) = 1.28, 95% CI = 1.02 to 1.62, *P* = 0.036), and the result was validated by MR-Egger (OR = 4.51, 95% CI = 1.26 to 16.19, *P* = 0.024, Table 2). We also verified the correctness of the inferred causal direction using the MR Steiger test for directionality (*P* < 0.001).

**Table 2:** MR results for the relationship between GRED and IPF in discovery cohort

Method	Number of SNPs	*β* / HR / OR	*P*-value
**Susceptibility**		**OR (95% CI)**	
IVW	75	1.28 (1.02–1.62)	0.036
Weighted median		1.13 (0.83–1.54)	0.442
MR-Egger		4.51 (1.26–16.19)	0.024
Simple mode		0.95 (0.43–2.11)	0.893
Weighted mode		1.04 (0.50–2.16)	0.923
**Susceptibility (excluded confounding factors)**		**OR (95% CI)**	
IVW	63	1.21 (0.95–1.55)	0.124
Weighted median		1.07 (0.77–1.49)	0.692
MR-Egger		3.87 (1.06–14.13)	0.045
Simple mode		0.90 (0.38–2.09)	0.802
Weighted mode		0.98 (0.46–2.08)	0.962
**FVC**		***β*** **± SE**	
IVW	62	26.63 ± 48.23	0.581
Weighted median		0.00 ± 71.77	1.000
MR-Egger		−113.24 ± 296.19	0.704
Simple mode		−30.29 ± 168.74	0.858
Weighted mode		−30.29 ± 173.48	0.862
**DLco**		***β*** **± SE**	
IVW	61	0.12 ± 0.12	0.319
Weighted median		0.06 ± 0.16	0.732
MR-Egger		−0.05 ± 0.75	0.948
Simple mode		0.80 ± 0.47	0.095
Weighted mode		−0.25 ± 0.38	0.521
**TFS**		**HR (95% CI)**	
IVW	60	0.87 (0.56–1.35)	0.533
Weighted median		0.72 (0.39–1.32)	0.286
MR-Egger		5.34 (0.42–67.80)	0.201
Simple mode		0.47 (0.09–2.33)	0.359
Weighted mode		0.47 (0.10–2.21)	0.343

*Abbreviations*: *MR* Mendelian randomization, *GERD* gastroesophageal reflux disease, *IPF* idiopathic pulmonary fibrosis, *SNP* single nucleotide polymorphism, *HR* hazard ratio, *OR* odds ratio, *SE* standard error, *CI* confidence intervals, *IVW* inverse variance weighted, *FVC* forced vital capacity, *DLco* diffusing capacity of the lung for carbon monoxide, *TFS* transplantation-free survival

**Fig. 2:**
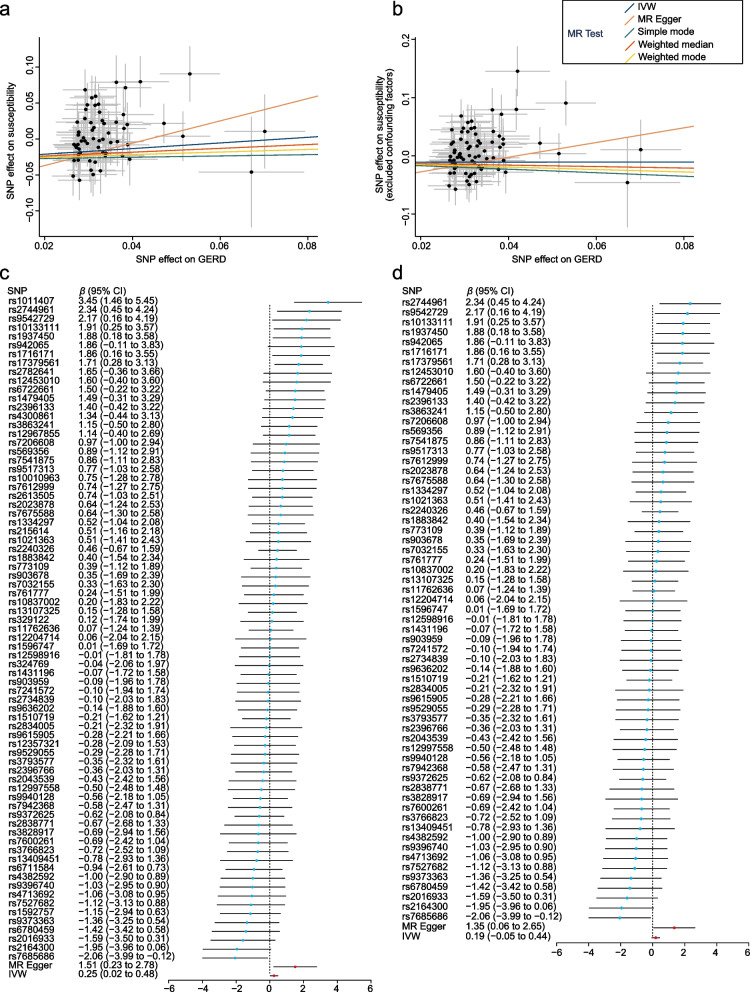
Scatterplots and forest plots of associations between GERD-associated SNPs and susceptibility to IPF in discovery cohort. Scatterplots of SNP effects on GERD and susceptibility to IPF before removing SNPs related to confounding factors and outliers (**a**), and after removal (**b**). Forest plots of individual and combined SNP MR-estimated effect size for GERD on the susceptibility to IPF before removing SNPs related to confounding factors and outliers (**c**), and after removal (**d**). Abbreviations: MR: Mendelian randomization; IVW: inverse variance weighted; GERD: gastroesophageal reflux disease; IPF: idiopathic pulmonary fibrosis; SNP: single nucleotide polymorphism

After removing SNPs related to confounding factors, we obtained 63 SNPs (Tables S2 and 3). The results of the IVW method showed no causal effect of GERD on susceptibility to IPF (OR = 1.21, 95% CI = 0.95 to 1.55, *P* = 0.124), and the result was also supported by weighted median, simple mode, and weighted mode (all *P* > 0.05, Table 2). Scatterplots and forest plots illustrating the associations between GERD-associated SNPs and susceptibility to IPF are presented in Fig. 2.

In the replication cohort, we obtained 9 SNPs as IVs to assess the genetic association of GERD with susceptibility to IPF (Tables S2 and S3), and no confounding factors were observed (Tables S1). The results of the IVW method revealed a causal effect of GERD on susceptibility to IPF (OR = 0.62, 95% CI = 0.40 to 0.97, *P* = 0.038, Table 3; Fig. S1). To verify the correctness of the inferred causal direction, we also conducted the MR Steiger test for directionality (*P* < 0.001).

**Table 3:** MR results for the relationship between GRED and IPF in replication cohort

Method	Number of SNPs	*β* / HR / OR	*P*-value
**Susceptibility**		**OR (95% CI)**	
IVW	9	0.62 (0.40–0.97)	0.038
Weighted median		0.61 (0.32–1.14)	0.122
MR-Egger		0.41 (0.14–1.22)	0.154
Simple mode		0.41 (0.15–1.16)	0.131
Weighted mode		0.44 (0.16–1.20)	0.146
**FVC**		***β*** **± SE**	
IVW	7	−72.38 ± 92.38	0.433
Weighted median		−68.91 ± 118.20	0.560
MR-Egger		− 144.95 ± 238.50	0.570
Simple mode		−32.10 ± 149.83	0.837
Weighted mode		−74.85 ± 170.92	0.677
**DLco**		***β*** **± SE**	
IVW	7	0.30 ± 0.22	0.169
Weighted median		0.56 ± 0.30	0.059
MR-Egger		−0.11 ± 0.61	0.869
Simple mode		0.70 ± 0.47	0.184
Weighted mode		0.68 ± 0.47	0.198
**TFS**		**HR (95% CI)**	
IVW	7	0.93 (0.44–1.97)	0.855
Weighted median		0.84 (0.33–2.15)	0.721
MR-Egger		0.98 (0.26–3.74)	0.979
Simple mode		1.20 (0.33–4.41)	0.791
Weighted mode		0.76 (0.23–2.50)	0.669

*Abbreviations*: *MR* Mendelian randomization, *GERD* gastroesophageal reflux disease, *IPF* idiopathic pulmonary fibrosis, *SNP* single nucleotide polymorphism, *HR* hazard ratio, *OR* odds ratio, *SE* standard error, *CI* confidence intervals, *IVW* inverse variance weighted, *FVC* forced vital capacity, *DLco* diffusing capacity of the lung for carbon monoxide, *TFS* transplantation-free survival

Furthermore, no statistically significant heterogeneity and horizontal pleiotropy was observed (Table S4). The MR-PRESSO global test, leave-one-out analysis, and funnel plots also provided no indications of any SNP outliers (Table S4; Figs. S2 and 3).

### Multivariable MR analysis adjusting for smoking initiation

We examined the effect of GERD on susceptibility to IPF while adjusting for smoking using multivariable MR analysis. The IVW method results indicated no causal effect of GERD on susceptibility to IPF after adjusting for smoking initiation (OR = 1.30, 95% CI = 0.93 to 1.68, *P* = 0.071), and this finding was supported by MVMR-Egger (OR = 0.91, 95% CI = 0.34 to 1.48, *P* = 0.767). These results were consistent in the replication cohort (all *P* > 0.05, Tables S5 and S6).

### Effect of GERD on the prognosis of IPF

**Fig. 3:**
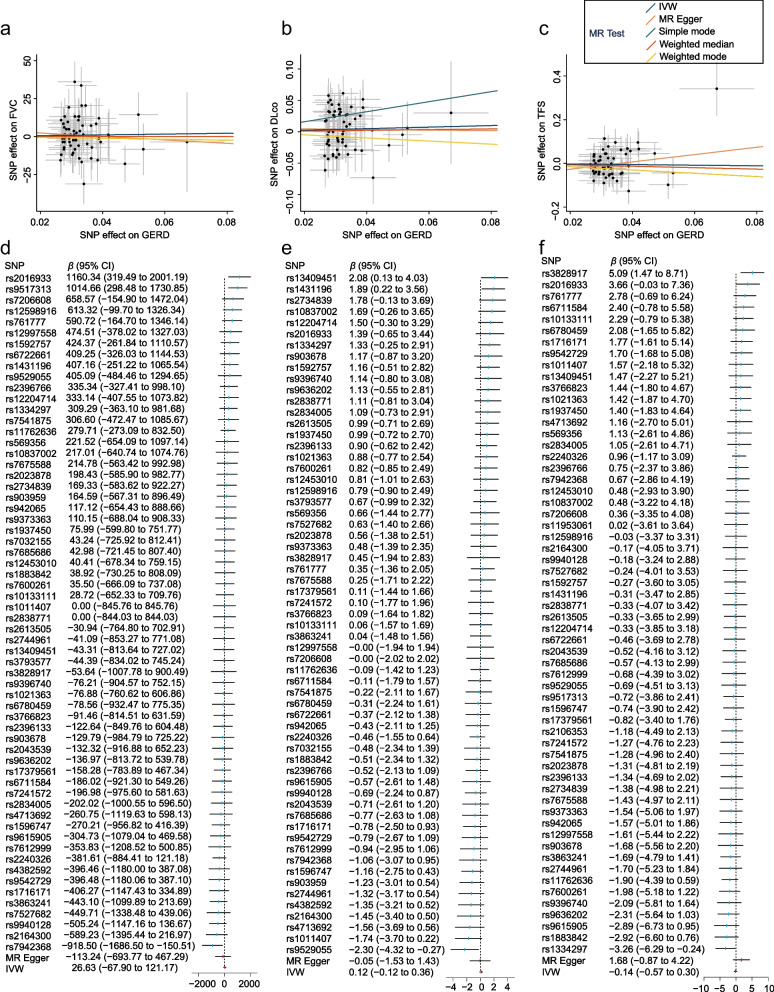
Scatterplots and forest plots of associations between GERD-associated SNPs and the prognosis of IPF in discovery cohort. Scatterplots of SNP effects on GERD and FVC (**a**), DLco (**b**), and TFS (**c**) in patients with IPF. Forest plots of individual and combined SNP MR-estimated effect size for GERD on FVC (**d**), DLco (**e**), and TFS (**f**). Abbreviations: MR: Mendelian randomization; IVW: inverse variance weighted; GERD: gastroesophageal reflux disease; FVC: forced vital capacity; DLco: diffusing capacity of the lung for carbon monoxide; TFS: transplantation-free survival; SNP: single nucleotide polymorphism

We obtained 62, 61, and 60 SNPs as IVs to assess the causal effect of GERD on FVC, DLco, and TFS, respectively (Tables S2 and S3). The results of the IVW method showed no causal effect of GERD on FVC (coefficient estimates (*β*) = 26.63, standard errors (SE) = 48.23, *P* = 0.581), DLco (*β* = 0.12, SE = 0.12, *P* = 0.319), and TFS (hazard ratio (HR) = 0.87, 95% confidence interval (CI) = 0.56 to 1.35, *P* = 0.533). Additionally, the results were validated by MR-Egger, weighted median, simple mode, and weighted mode (all *P* > 0.05, Table 2). Scatterplots and forest plots of associations between GERD-associated SNPs and FVC, DLco, and TFS in patients with IPF are presented in Fig. 3. In the replication cohort, we obtained 7 SNPs each as IVs to assess the causal effect of GERD on FVC, DLco, and TFS, respectively, and replicated these conclusions consistently (all *P* > 0.05, Table 3; Fig. S1).

Additionally, no statistically significant heterogeneity and horizontal pleiotropy was observed (Table S4). The MR-PRESSO global test, leave-one-out analysis, and funnel plots did not reveal any SNP outliers (Table S4; Figs. S3 and S4).

## Discussion

We conducted an investigation to explore the causal associations between GERD and the susceptibility and prognosis of IPF. In our study, after adjusting for smoking, we found no evidence that GERD increases susceptibility to IPF. Furthermore, our genetic evidence demonstrates no causal impact of GERD on FVC, DLco, and TFS in patients with IPF.

Similar to previous studies, initially we did not exclude SNPs associated with confounding factors, primarily those related to smoking (e.g., rs215614, rs12357321, rs329122, rs324769, and rs12967855). Consequently, we initially arrived at a similar conclusion to previous studies, suggesting a causal effect of GERD on susceptibility to IPF [13, 47]. However, Zhu, J et al. performed a multivariable MR and demonstrated that there is no causal effect of GERD on susceptibility to IPF after adjusting for smoking in a replicate cohort [47]. Smoking has been shown to have a causal relationship with an increased susceptibility to both IPF and GERD [11, 12]. Therefore, in the present study, smoking was included as potential confounder, and the results indicated no causal effect of GERD on susceptibility to IPF after adjusting for smoking.

Previous studies have suggested that GERD is an important risk factor for IPF, as gastroesophageal reflux has been reported in 76–94% of patients with IPF [48, 49]. Therefore, recent studies have explored the potential role of antacid medication in halting the progression of IPF [7, 8, 10, 50–54]. However, the majority of research did not yield the expected results [10, 50, 51, 53, 54], and two meta-analyses demonstrated that antacid medication had no statistically significant effect on arresting the disease progression of IPF [9, 10]. Therefore, guidelines do not recommend antacid medication and other interventions for improving respiratory outcomes in IPF [5]. Our study revealed no causal effect of GERD on FVC, DLco, and TFS in patients with IPF, providing some support for the recommendations outlined in the guidelines.

Anti-reflux surgery is designed to prevent both acid and non-acid refluxate. A prospective, randomized controlled trial was conducted to compare the decline in FVC between patients with IPF who underwent the surgery and those who did not. The study included 58 patients, and it was observed that the surgical group experienced a slower decline in FVC over a 48-week period (0.05 L) compared to the non-surgical group (0.13 L), but the difference did not reach statistical significance (*P* = 0.28) [55]. After conducting a recent meta-analysis of case-control studies, it has been suggested that the association between IPF and GERD may not stem from a direct causal relationship. Instead, it could be influenced by confounding factors, particularly smoking [56]. Combining our results, the recommendation to universally treat GERD in patients with IPF is further called into question.

The greatest strength of this study is its consideration of smoking and smoking-related SNPs in the MR analysis to examine the causal relationship between GERD and the susceptibility of IPF. The demonstrated absence of a causal relationship is attributed to the adjustment for smoking. Additionally, our findings suggest that there is no causal effect of GERD on FVC, DLco, or TFS in IPF. These results provide insights into the treatment options for IPF, indicating that the administration of universally recommended GERD therapy in the patients with IPF may not be supported.

This study has several limitations that should be acknowledged. First, the relatively small sample size in both the IPF GWAS (discovery and replication cohorts) and GERD GWAS (replication cohort) limited the precision of population parameter estimates, leading to larger standard errors. However, it’s noteworthy that these sample sizes were the largest ever used for these specific research questions. Second, we observed a relatively small number of significantly associated genetic loci for GERD within the replication cohort. The limited number of patients in the GERD replication cohort could have played a role in this restriction of significant loci, possibly leading to potential false-negative findings. Third, further investigations among populations with diverse racial and ethnic backgrounds are necessary, as the GWAS predominantly includes individuals of European ancestry. Therefore, caution must be exercised when generalizing the results to other populations.

## Conclusions

This study employed large GWAS data for an MR investigation into the relationship between GERD and susceptibility, FVC, DLco, and TFS of IPF. Our findings suggest that the association of GERD with susceptibility to IPF may not be directly causal and could be explained by confounding factors, particularly smoking. Furthermore, no observed causal effect of GERD on FVC, DLco, and TFS of IPF was found.

### Supplementary Information

**Additional file 1.**

## Data Availability

The datasets supporting the conclusions of this article are included within the article (and its Additional file 1).

## Abbreviations

IPF: Idiopathic pulmonary fibrosis
GWAS: Genome-wide association studies
GERD: Gastroesophageal reflux disease
MR: Mendelian randomization
MVMR: Multivariable Mendelian randomization
IVs: Instrumental variables
FVC: Forced vital capacity
DLco: Diffusing capacity of the lung for carbon monoxide
TFS: Transplant-free survival
SNP: Single nucleotide polymorphism
IVW: Inverse variance weighted
*β*: Coefficient estimates
SE: Standard errors
95% CI: 95% Confidence interval
OR: Odds ratio
