# Supplementary material for: Mendelian randomization analysis suggests no causal influence of gastroesophageal reflux disease on the susceptibility and prognosis of idiopathic pulmonary fibrosis
Source: BMC Pulm Med. 2023 Dec 21;23:517. doi: 10.1186/s12890-023-02788-8 (PMC10740234; doi:10.1186/s12890-023-02788-8)
Supplement: Supplementary file 1 — Additional file 1. [file 12890_2023_2788_MOESM1_ESM.pdf]

Supplemental Material

Mendelian Randomization Analysis Suggests No Causal Influence of Gastroesophageal Reflux Disease on the Susceptibility and Prognosis of Idiopathic Pulmonary Fibrosis

Di Sun, Qiao Ye*

Department of Occupational Medicine and Toxicology, Clinical Center for Interstitial Lung Diseases, Beijing Institute of Respiratory Medicine, Beijing Chao-Yang Hospital, Capital Medical University, Beijing 100020, China

* Correspondence:

Professor Qiao Ye

Department of Occupational Medicine and Toxicology, Clinical Center for Interstitial Lung Diseases, Beijing Institute of Respiratory Medicine, Beijing Chao-Yang Hospital, Capital Medical University

yeqiao_chaoyang@sina.com

Table S1 Potential confounders of exposure SNPs in the PhenoScanner database

Exposure	Outcomes	Excluded SNPs	Trait
GERD (discovery cohort)	FVC	rs215614	Smoking behavior
		rs13107325	FEV_1% predicted
		rs12357321	FVC
		rs2782641	Smoking behavior
		rs12967855	Smoking behavior, FEV_1, FVC
		rs1510719	FEV_1
		rs1479405	FEV_1
		rs10010963	Smoking behavior
		rs773109	FEV_1
		rs9372625	Exposure to tobacco smoke, FEV_1, FVC
		rs329122	Smoking behavior
		rs324769	Smoking behavior
		rs4300861	Smoking behavior
	DLCO	rs13107325	FEV_1% predicted
		rs12357321	FVC
		rs2782641	Smoking behavior
		rs12967855	Smoking behavior, FEV_1, FVC
		rs1510719	FEV_1
		rs1479405	FEV_1
		rs10010963	Smoking behavior
		rs773109	FEV_1
		rs9372625	Exposure to tobacco smoke, FEV_1, FVC
		rs329122	Smoking behavior
		rs324769	Smoking behavior
		rs4300861	Smoking behavior
	TFS	rs215614	Smoking behavior
		rs13107325	FEV_1% predicted
		rs12357321	FVC
		rs12967855	Smoking behavior, FEV_1, FVC
		rs1510719	FEV_1
		rs1479405	FEV_1
		rs10010963	Smoking behavior
		rs773109	FEV_1
		rs9372625	Exposure to tobacco smoke, FEV_1, FVC
		rs2782641	Smoking behavior
		rs329122	Smoking behavior
		rs4300861	Smoking behavior
		rs324769	Smoking behavior
	Susceptibility	rs215614	Smoking behavior
		rs2613505	Smoking behavior, previous
		rs2782641	Smoking behavior

Exposure	Outcomes	Excluded SNPs	Trait
		rs12357321	Smoking behavior, previous
		rs1592757	Smoking behavior, previous
		rs10010963	Smoking behavior
		rs6711584	Smoking behavior, previous
		rs329122	Smoking behavior
		rs324769	Smoking behavior
		rs4300861	Smoking behavior
		rs12967855	Smoking behavior
GERD (replication cohort)	FVC	rs10887132	FEV1, FVC
	DLCO	rs10887132	FEV1, FVC
	TFS	rs10887132	FEV1, FVC
	Susceptibility	None	-

The potential confounders of FVC, DLco, and TFS in patients with idiopathic pulmonary fibrosis included pulmonary function testing, pollution and occupation (excluded previous exposures), and pulmonary infection. The potential confounders of susceptibility on idiopathic pulmonary fibrosis included pollution and occupation, animal antigens, and viral exposures. Abbreviations: SNP: single nucleotide polymorphism; GERD: gastroesophageal reflux disease; FVC: forced vital capacity; FEV_1: forced expiratory volume in 1 s; DLco: diffuse lung capacity for carbon monoxide; TFS: transplantation-free survival.

Table S2 Summary information on SNPs of GRED used as genetic instruments for the MR analyses

SNPs	EA	NEA	EAF	Beta	SE	Sample size	*P*-value	R^2	*F*-statistics
**62 SNPs for GRED on FVC, discovery cohort**									
rs1011407	A	G	0.878	0.042	0.007	55,499	1.09×10^{-8}	3.78×10^{-4}	33
rs10133111	G	A	0.837	-0.042	0.007	55,581	1.35×10^{-10}	$4.76 \times 10^{-}$	41
rs1021363	A	G	0.358	0.031	0.005	55,401	5.10×10^{-10}	4.48×10^{-4}	39
rs10837002	C	G	0.649	-0.028	0.005	55,605	4.03×10^{-8}	3.48×10^{-4}	30
rs11762636	C	A	0.820	0.051	0.006	55,423	1.88×10^{-16}	7.83×10^{-4}	68
rs12204714	C	T	0.368	0.029	0.005	55,429	7.92×10^{-9}	3.86×10^{-4}	33
rs12453010	C	T	0.605	-0.030	0.005	55,027	1.75×10^{-9}	4.21×10^{-4}	36
rs12598916	C	G	0.725	0.033	0.005	55,368	6.87×10^{-10}	4.41×10^{-4}	38
rs12997558	G	A	0.641	-0.028	0.005	55,346	3.04×10^{-8}	3.56×10^{-4}	31
rs1334297	G	A	0.266	0.039	0.005	55,300	1.14×10^{-12}	5.87×10^{-4}	51
rs13409451	A	G	0.608	0.028	0.005	55,257	1.93×10^{-8}	3.66×10^{-4}	32
rs1431196	A	G	0.572	-0.032	0.005	55,140	2.65×10^{-11}	5.14×10^{-4}	44
rs1592757	G	C	0.644	-0.031	0.005	55,559	6.00×10^{-10}	4.44×10^{-4}	38
rs1596747	A	G	0.506	-0.031	0.005	55,670	1.00×10^{-10}	4.83×10^{-4}	42
rs1716171	C	T	0.210	-0.038	0.006	55,381	7.82×10^{-11}	4.89×10^{-4}	42
rs17379561	A	T	0.856	-0.053	0.007	55,522	1.08×10^{-14}	6.96×10^{-4}	60
rs1883842	T	G	0.721	-0.031	0.005	55,209	9.27×10^{-9}	3.83×10^{-4}	33
rs1937450	T	G	0.462	-0.032	0.005	54,639	7.07×10^{-11}	4.96×10^{-4}	43
rs2016933	C	G	0.270	0.031	0.005	55,414	1.04×10^{-8}	3.79×10^{-4}	33
rs2023878	C	T	0.808	0.036	0.006	55,227	3.04×10^{-9}	4.09×10^{-4}	35
rs2043539	G	A	0.581	-0.027	0.005	55,631	2.24×10^{-8}	3.60×10^{-4}	31
rs2164300	C	T	0.477	0.026	0.005	55,075	4.13×10^{-8}	3.50×10^{-4}	30

SNPs	EA	NEA	EAF	Beta	SE	Sample size	*P*-value	R^2	*F*-statistics
rs2240326	G	A	0.526	0.047	0.005	55,674	1.13×10^{-22}	1.11×10^{-3}	96
rs2396133	A	G	0.525	-0.029	0.005	55,446	1.11×10^{-9}	4.30×10^{-4}	37
rs2396766	G	A	0.527	-0.032	0.005	55,425	2.33×10^{-11}	5.17×10^{-4}	45
rs2613505	C	T	0.200	-0.039	0.006	55,418	1.17×10^{-10}	4.80×10^{-4}	42
rs2734839	C	T	0.393	0.028	0.005	55,355	8.79×10^{-9}	3.84×10^{-4}	33
rs2744961	C	T	0.642	-0.029	0.005	55,635	5.81×10^{-9}	3.92×10^{-4}	34
rs2834005	T	C	0.685	-0.030	0.005	55,500	9.42×10^{-9}	3.81×10^{-4}	33
rs2838771	G	C	0.353	0.028	0.005	55,271	2.91×10^{-8}	3.61×10^{-4}	31
rs3766823	G	A	0.829	-0.039	0.006	55,515	7.09×10^{-10}	4.40×10^{-4}	38
rs3793577	A	G	0.462	-0.027	0.005	54,460	2.49×10^{-8}	3.63×10^{-4}	31
rs3828917	G	T	0.958	-0.067	0.012	55,673	2.27×10^{-8}	3.61×10^{-4}	31
rs3863241	C	T	0.473	-0.032	0.005	55,578	1.49×10^{-11}	5.27×10^{-4}	46
rs4382592	T	G	0.300	0.030	0.005	55,446	8.20×10^{-9}	3.85×10^{-4}	33
rs4713692	C	T	0.632	0.028	0.005	55,721	3.07×10^{-8}	3.55×10^{-4}	31
rs569356	A	G	0.859	0.038	0.007	55,601	4.07×10^{-8}	3.48×10^{-4}	30
rs6711584	G	A	0.548	-0.032	0.005	55,140	2.66×10^{-11}	5.15×10^{-4}	44
rs6722661	G	A	0.635	0.032	0.005	55,396	1.15×10^{-10}	4.82×10^{-4}	42
rs6780459	A	T	0.253	-0.031	0.006	55,576	3.14×10^{-8}	3.53×10^{-4}	31
rs7032155	C	A	0.408	-0.028	0.005	54,863	1.63×10^{-8}	3.72×10^{-4}	32
rs7206608	C	G	0.677	-0.029	0.005	55,434	1.46×10^{-8}	3.72×10^{-4}	32
rs7241572	G	A	0.791	-0.037	0.006	53,887	9.49×10^{-10}	4.42×10^{-4}	37
rs7527682	A	G	0.463	0.027	0.005	55,547	3.13×10^{-8}	3.54×10^{-4}	31
rs7541875	A	G	0.574	-0.027	0.005	55,810	1.61×10^{-8}	3.67×10^{-4}	32
rs7600261	C	T	0.694	-0.034	0.005	55,531	9.47×10^{-11}	4.86×10^{-4}	42

SNPs	EA	NEA	EAF	Beta	SE	Sample size	*P*-value	R^2	*F*-statistics
rs7612999	G	A	0.755	-0.031	0.006	55,396	4.90×10^{-8}	3.45×10^{-4}	30
rs761777	A	G	0.746	-0.035	0.006	55,606	4.71×10^{-10}	4.52×10^{-4}	39
rs7675588	C	A	0.205	0.034	0.006	55,455	1.80×10^{-8}	3.67×10^{-4}	32
rs7685686	A	G	0.578	0.028	0.005	55,432	1.14×10^{-8}	3.80×10^{-4}	33
rs7942368	C	T	0.785	0.034	0.006	53,991	9.54×10^{-9}	3.89×10^{-4}	33
rs903678	G	A	0.661	-0.028	0.005	55,569	4.89×10^{-8}	3.45×10^{-4}	30
rs903959	T	A	0.601	-0.029	0.005	55,339	2.99×10^{-9}	4.08×10^{-4}	35
rs9373363	A	G	0.746	0.033	0.006	54,713	4.13×10^{-9}	4.04×10^{-4}	35
rs9396740	G	A	0.751	0.031	0.006	55,476	1.47×10^{-8}	3.71×10^{-4}	32
rs942065	G	A	0.366	-0.031	0.005	55,098	8.45×10^{-10}	4.38×10^{-4}	38
rs9517313	G	C	0.617	-0.033	0.005	55,658	2.05×10^{-11}	5.18×10^{-4}	45
rs9529055	G	A	0.524	-0.027	0.005	55,506	3.11×10^{-8}	3.55×10^{-4}	31
rs9542729	C	G	0.798	0.036	0.006	55,285	1.41×10^{-9}	4.26×10^{-4}	37
rs9615905	C	T	0.542	-0.028	0.005	55,538	1.21×10^{-8}	3.77×10^{-4}	32
rs9636202	G	A	0.733	0.035	0.005	55,069	1.51×10^{-10}	4.80×10^{-4}	41
rs9940128	G	A	0.578	-0.033	0.005	55,566	8.06×10^{-12}	5.39×10^{-4}	47
**61 SNPs for GRED on DLco, discovery cohort**									
rs1011407	A	G	0.878	0.042	0.007	55499	1.09×10^{-8}	3.78×10^{-4}	33
rs10133111	G	A	0.837	-0.042	0.007	55581	1.35×10^{-10}	4.76×10^{-4}	41
rs1021363	A	G	0.358	0.031	0.005	55401	5.10×10^{-10}	4.48×10^{-4}	39
rs10837002	C	G	0.649	-0.028	0.005	55605	4.03×10^{-8}	3.48×10^{-4}	30
rs11762636	C	A	0.820	0.051	0.006	55423	1.88×10^{-16}	7.83×10^{-4}	68
rs12204714	C	T	0.368	0.029	0.005	55429	7.92×10^{-9}	3.86×10^{-4}	33
rs12453010	C	T	0.605	-0.030	0.005	55027	1.75×10^{-9}	4.21×10^{-4}	36

SNPs	EA	NEA	EAF	Beta	SE	Sample size	*P*-value	R^2	*F*-statistics
rs12598916	C	G	0.725	0.033	0.005	55368	6.87×10^{-10}	4.41×10^{-4}	38
rs12997558	G	A	0.641	-0.028	0.005	55,346	3.04×10^{-8}	3.56×10^{-4}	31
rs1334297	G	A	0.266	0.039	0.005	55,300	1.14×10^{-12}	5.87×10^{-4}	51
rs13409451	A	G	0.608	0.028	0.005	55,257	1.93×10^{-8}	3.66×10^{-4}	32
rs1431196	A	G	0.572	-0.032	0.005	55,140	2.65×10^{-11}	5.15×10^{-4}	44
rs1592757	G	C	0.644	-0.031	0.005	55,559	6.00×10^{-10}	4.44×10^{-4}	38
rs1596747	A	G	0.506	-0.031	0.005	55,670	1.00×10^{-10}	4.83×10^{-4}	42
rs1716171	C	T	0.210	-0.038	0.006	55,381	7.82×10^{-11}	4.89×10^{-4}	42
rs17379561	A	T	0.856	-0.053	0.007	55,522	1.08×10^{-14}	6.96×10^{-4}	60
rs1883842	T	G	0.721	-0.031	0.005	55,209	9.27×10^{-9}	3.83×10^{-4}	33
rs1937450	T	G	0.462	-0.032	0.005	54,639	7.07×10^{-11}	4.96×10^{-4}	43
rs2016933	C	G	0.270	0.031	0.005	55,414	1.04×10^{-8}	3.79×10^{-4}	33
rs2023878	C	T	0.808	0.036	0.006	55,227	3.04×10^{-9}	4.09×10^{-4}	35
rs2043539	G	A	0.581	-0.027	0.005	55,631	2.24×10^{-8}	3.60×10^{-4}	31
rs2164300	C	T	0.477	0.026	0.005	55,075	4.13×10^{-8}	3.50×10^{-4}	30
rs2240326	G	A	0.526	0.047	0.005	55,674	1.13×10^{-22}	1.11×10^{-3}	96
rs2396133	A	G	0.525	-0.029	0.005	55,446	1.11×10^{-9}	4.30×10^{-4}	37
rs2396766	G	A	0.527	-0.032	0.005	55,425	2.33×10^{-11}	5.17×10^{-4}	45
rs2613505	C	T	0.200	-0.039	0.006	55,418	1.17×10^{-10}	4.80×10^{-4}	42
rs2734839	C	T	0.393	0.028	0.005	55,355	8.79×10^{-9}	3.84×10^{-4}	33
rs2744961	C	T	0.642	-0.029	0.005	55,635	5.81×10^{-9}	3.92×10^{-4}	34
rs2834005	T	C	0.685	-0.030	0.005	55,500	9.42×10^{-9}	3.81×10^{-4}	33
rs2838771	G	C	0.353	0.028	0.005	55,271	2.91×10^{-8}	3.61×10^{-4}	31
rs3766823	G	A	0.829	-0.039	0.006	55,515	7.09×10^{-10}	4.40×10^{-4}	38

SNPs	EA	NEA	EAF	Beta	SE	Sample size	*P*-value	R^2	*F*-statistics
rs3793577	A	G	0.462	-0.027	0.005	54,460	2.49×10^{-8}	3.63×10^{-4}	31
rs3828917	G	T	0.958	-0.067	0.012	55,673	2.27×10^{-8}	3.61×10^{-4}	31
rs3863241	C	T	0.473	-0.032	0.005	55,578	1.49×10^{-11}	5.27×10^{-4}	46
rs4382592	T	G	0.300	0.030	0.005	55,446	8.20×10^{-9}	3.85×10^{-4}	33
rs4713692	C	T	0.632	0.028	0.005	55,721	3.07×10^{-8}	3.55×10^{-4}	31
rs569356	A	G	0.859	0.038	0.007	55,601	4.07×10^{-8}	3.48×10^{-4}	30
rs6711584	G	A	0.548	-0.032	0.005	55,140	2.66×10^{-11}	5.15×10^{-4}	44
rs6722661	G	A	0.635	0.032	0.005	55,396	1.15×10^{-10}	4.82×10^{-4}	42
rs6780459	A	T	0.253	-0.031	0.006	55,576	3.14×10^{-8}	3.53×10^{-4}	31
rs7032155	C	A	0.408	-0.028	0.005	54,863	1.63×10^{-8}	3.72×10^{-4}	32
rs7206608	C	G	0.677	-0.029	0.005	55,434	1.46×10^{-8}	3.72×10^{-4}	32
rs7241572	G	A	0.791	-0.037	0.006	53,887	9.49×10^{-10}	4.42×10^{-4}	37
rs7527682	A	G	0.463	0.027	0.005	55,547	3.13×10^{-8}	3.54×10^{-4}	31
rs7541875	A	G	0.574	-0.027	0.005	55,810	1.61×10^{-8}	3.67×10^{-4}	32
rs7600261	C	T	0.694	-0.034	0.005	55,531	9.47×10^{-11}	4.86×10^{-4}	42
rs7612999	G	A	0.755	-0.031	0.006	55,396	4.90×10^{-8}	3.45×10^{-4}	30
rs761777	A	G	0.746	-0.035	0.006	55,606	4.71×10^{-10}	4.52×10^{-4}	39
rs7675588	C	A	0.205	0.034	0.006	55,455	1.80×10^{-8}	3.67×10^{-4}	32
rs7685686	A	G	0.578	0.028	0.005	55,432	1.14×10^{-8}	3.80×10^{-4}	33
rs7942368	C	T	0.785	0.034	0.006	53,991	9.54×10^{-9}	3.89×10^{-4}	33
rs903678	G	A	0.661	-0.028	0.005	55,569	4.89×10^{-8}	3.45×10^{-4}	30
rs903959	T	A	0.601	-0.029	0.005	55,339	2.99×10^{-9}	4.08×10^{-4}	35
rs9373363	A	G	0.746	0.033	0.006	54,713	4.13×10^{-9}	4.04×10^{-4}	35
rs9396740	G	A	0.751	0.031	0.006	55,476	1.47×10^{-8}	3.71×10^{-4}	32

SNPs	EA	NEA	EAF	Beta	SE	Sample size	*P*-value	R^2	*F*-statistics
rs942065	G	A	0.366	-0.031	0.005	55,098	8.45×10^{-10}	4.38×10^{-4}	38
rs9529055	G	A	0.524	-0.027	0.005	55,506	3.11×10^{-8}	3.55×10^{-4}	31
rs9542729	C	G	0.798	0.036	0.006	55,285	1.41×10^{-9}	4.26×10^{-4}	37
rs9615905	C	T	0.542	-0.028	0.005	55,538	1.21×10^{-8}	3.77×10^{-4}	32
rs9636202	G	A	0.733	0.035	0.005	55,069	1.51×10^{-10}	4.80×10^{-4}	41
rs9940128	G	A	0.578	-0.033	0.005	55,566	8.06×10^{-12}	5.39×10^{-4}	47
**60 SNPs for GRED on TFS, discovery cohort**									
rs1011407	A	G	0.878	0.042	0.007	55,499	1.09×10^{-8}	3.78×10^{-4}	33
rs10133111	G	A	0.837	-0.042	0.007	55,581	1.35×10^{-10}	4.76×10^{-4}	41
rs1021363	A	G	0.358	0.031	0.005	55,401	5.10×10^{-10}	4.48×10^{-4}	39
rs10837002	C	G	0.649	-0.028	0.005	55,605	4.03×10^{-8}	3.48×10^{-4}	30
rs11762636	C	A	0.820	0.051	0.006	55,423	1.88×10^{-16}	7.83×10^{-4}	68
rs11953061	C	T	0.661	-0.028	0.005	55,084	3.10×10^{-8}	3.55×10^{-4}	31
rs12204714	C	T	0.368	0.029	0.005	55,429	7.92×10^{-9}	3.86×10^{-4}	33
rs12453010	C	T	0.605	-0.030	0.005	55,027	1.75×10^{-9}	4.21×10^{-4}	36
rs12598916	C	G	0.725	0.033	0.005	55,368	6.87×10^{-10}	4.41×10^{-4}	38
rs12997558	G	A	0.641	-0.028	0.005	55,346	3.04×10^{-8}	3.56×10^{-4}	31
rs1334297	G	A	0.266	0.039	0.005	55,300	1.14×10^{-12}	5.87×10^{-4}	51
rs13409451	A	G	0.608	0.028	0.005	55,257	1.93×10^{-8}	3.66×10^{-4}	32
rs1431196	A	G	0.572	-0.032	0.005	55,140	2.65×10^{-11}	5.15×10^{-4}	44
rs1592757	G	C	0.644	-0.031	0.005	55,559	6.00×10^{-10}	4.44×10^{-4}	38
rs1596747	A	G	0.506	-0.031	0.005	55,670	1.00×10^{-10}	4.83×10^{-4}	42
rs1716171	C	T	0.210	-0.038	0.006	55,381	7.82×10^{-11}	4.89×10^{-4}	42
rs17379561	A	T	0.856	-0.053	0.007	55,522	1.08×10^{-14}	6.96×10^{-4}	60

SNPs	EA	NEA	EAF	Beta	SE	Sample size	*P*-value	R^2	*F*-statistics
rs1883842	T	G	0.721	-0.031	0.005	55,209	9.27×10^{-9}	3.83×10^{-4}	33
rs1937450	T	G	0.462	-0.032	0.005	54,639	7.07×10^{-11}	4.96×10^{-4}	43
rs2016933	C	G	0.270	0.031	0.005	55,414	1.04×10^{-8}	3.79×10^{-4}	33
rs2023878	C	T	0.808	0.036	0.006	55,227	3.04×10^{-9}	4.09×10^{-4}	35
rs2043539	G	A	0.581	-0.027	0.005	55,631	2.24×10^{-8}	3.60×10^{-4}	31
rs2106353	G	T	0.769	-0.037	0.006	55,129	1.37×10^{-10}	4.80×10^{-4}	41
rs2164300	C	T	0.477	0.026	0.005	55,075	4.13×10^{-8}	3.50×10^{-4}	30
rs2240326	G	A	0.526	0.047	0.005	55,674	1.13×10^{-22}	1.11×10^{-3}	96
rs2396133	A	G	0.525	-0.029	0.005	55,446	1.11×10^{-9}	4.30×10^{-4}	37
rs2396766	G	A	0.527	-0.032	0.005	55,425	2.33×10^{-11}	5.17×10^{-4}	45
rs2613505	C	T	0.200	-0.039	0.006	55,418	1.17×10^{-10}	4.80×10^{-4}	42
rs2734839	C	T	0.393	0.028	0.005	55,355	8.79×10^{-9}	3.84×10^{-4}	33
rs2744961	C	T	0.642	-0.029	0.005	55,635	5.81×10^{-9}	3.92×10^{-4}	34
rs2834005	T	C	0.685	-0.030	0.005	55,500	9.42×10^{-9}	3.81×10^{-4}	33
rs2838771	G	C	0.353	0.028	0.005	55,271	2.91×10^{-8}	3.61×10^{-4}	31
rs3766823	G	A	0.829	-0.039	0.006	55,515	7.09×10^{-10}	4.40×10^{-4}	38
rs3828917	G	T	0.958	-0.067	0.012	55,673	2.27×10^{-8}	3.61×10^{-4}	31
rs3863241	C	T	0.473	-0.032	0.005	55,578	1.49×10^{-11}	5.27×10^{-4}	46
rs4713692	C	T	0.632	0.028	0.005	55,721	3.07×10^{-8}	3.55×10^{-4}	31
rs569356	A	G	0.859	0.038	0.007	55,601	4.07×10^{-8}	3.48×10^{-4}	30
rs6711584	G	A	0.548	-0.032	0.005	55,140	2.66×10^{-11}	5.15×10^{-4}	44
rs6722661	G	A	0.635	0.032	0.005	55,396	1.15×10^{-10}	4.82×10^{-4}	42
rs6780459	A	T	0.253	-0.031	0.006	55,576	3.14×10^{-8}	3.53×10^{-4}	31
rs7206608	C	G	0.677	-0.029	0.005	55,434	1.46×10^{-8}	3.72×10^{-4}	32

SNPs	EA	NEA	EAF	Beta	SE	Sample size	*P*-value	R^2	*F*-statistics
rs7241572	G	A	0.791	-0.037	0.006	53,887	9.49×10^{-10}	4.42×10^{-4}	37
rs7527682	A	G	0.463	0.027	0.005	55,547	3.13×10^{-8}	3.54×10^{-4}	31
rs7541875	A	G	0.574	-0.027	0.005	55,810	1.61×10^{-8}	3.67×10^{-4}	32
rs7600261	C	T	0.694	-0.034	0.005	55,531	9.47×10^{-11}	4.86×10^{-4}	42
rs7612999	G	A	0.755	-0.031	0.006	55,396	4.90×10^{-8}	3.45×10^{-4}	30
rs761777	A	G	0.746	-0.035	0.006	55,606	4.71×10^{-10}	4.52×10^{-4}	39
rs7675588	C	A	0.205	0.034	0.006	55,455	1.80×10^{-8}	3.67×10^{-4}	32
rs7685686	A	G	0.578	0.028	0.005	55,432	1.14×10^{-8}	3.80×10^{-4}	33
rs7942368	C	T	0.785	0.034	0.006	53,991	9.54×10^{-9}	3.89×10^{-4}	33
rs903678	G	A	0.661	-0.028	0.005	55,569	4.89×10^{-8}	3.45×10^{-4}	30
rs9373363	A	G	0.746	0.033	0.006	54,713	4.13×10^{-9}	4.04×10^{-4}	35
rs9396740	G	A	0.751	0.031	0.006	55,476	1.47×10^{-8}	3.71×10^{-4}	32
rs942065	G	A	0.366	-0.031	0.005	55,098	8.45×10^{-10}	4.38×10^{-4}	38
rs9517313	G	C	0.617	-0.033	0.005	55,658	2.05×10^{-11}	5.18×10^{-4}	45
rs9529055	G	A	0.524	-0.027	0.005	55,506	3.11×10^{-8}	3.55×10^{-4}	31
rs9542729	C	G	0.798	0.036	0.006	55,285	1.41×10^{-9}	4.26×10^{-4}	37
rs9615905	C	T	0.542	-0.028	0.005	55,538	1.21×10^{-8}	3.77×10^{-4}	32
rs9636202	G	A	0.733	0.035	0.005	55,069	1.51×10^{-10}	4.80×10^{-4}	41
rs9940128	G	A	0.578	-0.033	0.005	55,566	8.06×10^{-12}	5.39×10^{-4}	47
**75 SNPs for GRED on susceptibility, discovery cohort**									
rs10010963	C	T	0.384	0.027	0.005	55,391	4.92×10^{-8}	3.44×10^{-4}	30
rs1011407	A	G	0.878	0.042	0.007	55,499	1.09×10^{-8}	3.78×10^{-4}	33
rs10133111	G	A	0.837	-0.042	0.007	55,581	1.35×10^{-10}	4.76×10^{-4}	41
rs1021363	A	G	0.358	0.031	0.005	55,401	5.10×10^{-10}	4.48×10^{-4}	39

SNPs	EA	NEA	EAF	Beta	SE	Sample size	*P*-value	R^2	*F*-statistics
rs10837002	C	G	0.649	-0.028	0.005	55,605	4.03×10^{-8}	3.48×10^{-4}	30
rs11762636	C	A	0.820	0.051	0.006	55,423	1.88×10^{-16}	7.83×10^{-4}	68
rs12204714	C	T	0.368	0.029	0.005	55,429	7.92×10^{-9}	3.86×10^{-4}	33
rs12357321	G	A	0.689	-0.032	0.005	54,582	1.33×10^{-9}	4.31×10^{-4}	37
rs12453010	C	T	0.605	-0.030	0.005	55,027	1.75×10^{-9}	4.21×10^{-4}	36
rs12598916	C	G	0.725	0.033	0.005	55,368	6.87×10^{-10}	4.41×10^{-4}	38
rs12967855	A	G	0.330	0.037	0.005	55,051	1.09×10^{-12}	5.90×10^{-4}	51
rs12997558	G	A	0.641	-0.028	0.005	55,346	3.04×10^{-8}	3.56×10^{-4}	31
rs13107325	C	T	0.926	-0.070	0.009	55,434	2.20×10^{-14}	6.78×10^{-4}	58
rs1334297	G	A	0.266	0.039	0.005	55,300	1.14×10^{-12}	5.87×10^{-4}	51
rs13409451	A	G	0.608	0.028	0.005	55,257	1.93×10^{-8}	3.66×10^{-4}	32
rs1431196	A	G	0.572	-0.032	0.005	55,140	2.65×10^{-11}	5.15×10^{-4}	44
rs1479405	C	T	0.678	-0.031	0.005	55,492	9.85×10^{-10}	4.33×10^{-4}	37
rs1510719	T	C	0.617	0.039	0.005	55,373	3.84×10^{-15}	7.15×10^{-4}	62
rs1592757	G	C	0.644	-0.031	0.005	55,559	6.00×10^{-10}	4.44×10^{-4}	38
rs1596747	A	G	0.506	-0.031	0.005	55,670	1.00×10^{-10}	4.83×10^{-4}	42
rs1716171	C	T	0.210	-0.038	0.006	55,381	7.82×10^{-11}	4.89×10^{-4}	42
rs17379561	A	T	0.856	-0.053	0.007	55,522	1.08×10^{-14}	6.96×10^{-4}	60
rs1883842	T	G	0.721	-0.031	0.005	55,209	9.27×10^{-9}	3.83×10^{-4}	33
rs1937450	T	G	0.462	-0.032	0.005	54,639	7.07×10^{-11}	4.96×10^{-4}	43
rs2016933	C	G	0.270	0.031	0.005	55,414	1.04×10^{-8}	3.79×10^{-4}	33
rs2023878	C	T	0.808	0.036	0.006	55,227	3.04×10^{-9}	4.09×10^{-4}	35
rs2043539	G	A	0.581	-0.027	0.005	55,631	2.24×10^{-8}	3.60×10^{-4}	31
rs215614	G	A	0.370	0.033	0.005	55,406	4.08×10^{-11}	5.03×10^{-4}	44

**SNPs**	**EA**	**NEA**	**EAF**	**Beta**	**SE**	**Sample size**	***P*-value**	**R^2**	***F*-statistics**
rs2164300	C	T	0.477	0.026	0.005	55,075	4.13×10^{-8}	3.50×10^{-4}	30
rs2240326	G	A	0.526	0.047	0.005	55,674	1.13×10^{-22}	1.11×10^{-3}	96
rs2396133	A	G	0.525	-0.029	0.005	55,446	1.11×10^{-9}	4.30×10^{-4}	37
rs2396766	G	A	0.527	-0.032	0.005	55,425	2.33×10^{-11}	5.17×10^{-4}	45
rs2613505	C	T	0.200	-0.039	0.006	55,418	1.17×10^{-10}	4.80×10^{-4}	42
rs2734839	C	T	0.393	0.028	0.005	55,355	8.79×10^{-9}	3.84×10^{-4}	33
rs2744961	C	T	0.642	-0.029	0.005	55,635	5.81×10^{-9}	3.92×10^{-4}	34
rs2782641	G	A	0.387	-0.027	0.005	55,107	4.33×10^{-8}	3.48×10^{-4}	30
rs2834005	T	C	0.685	-0.030	0.005	55,500	9.42×10^{-9}	3.81×10^{-4}	33
rs2838771	G	C	0.353	0.028	0.005	55,271	2.91×10^{-8}	3.61×10^{-4}	31
rs324769	C	T	0.551	0.027	0.005	55,497	3.05×10^{-8}	3.55×10^{-4}	31
rs329122	G	A	0.580	0.029	0.005	55,403	3.05×10^{-9}	4.08×10^{-4}	35
rs3766823	G	A	0.829	-0.039	0.006	55,515	7.09×10^{-10}	4.40×10^{-4}	38
rs3793577	A	G	0.462	-0.027	0.005	54,460	2.49×10^{-8}	3.63×10^{-4}	31
rs3828917	G	T	0.958	-0.067	0.012	55,673	2.27×10^{-8}	3.61×10^{-4}	31
rs3863241	C	T	0.473	-0.032	0.005	55,578	1.49×10^{-11}	5.27×10^{-4}	46
rs4300861	C	T	0.618	-0.031	0.005	55,421	5.43×10^{-10}	4.45×10^{-4}	39
rs4382592	T	G	0.300	0.030	0.005	55,446	8.20×10^{-9}	3.85×10^{-4}	33
rs4713692	C	T	0.632	0.028	0.005	55,721	3.07×10^{-8}	3.55×10^{-4}	31
rs569356	A	G	0.859	0.038	0.007	55,601	4.07×10^{-8}	3.48×10^{-4}	30
rs6711584	G	A	0.548	-0.032	0.005	55,140	2.66×10^{-11}	5.15×10^{-4}	44
rs6722661	G	A	0.635	0.032	0.005	55,396	1.15×10^{-10}	4.82×10^{-4}	42
rs6780459	A	T	0.253	-0.031	0.006	55,576	3.14×10^{-8}	3.53×10^{-4}	31
rs7032155	C	A	0.408	-0.028	0.005	54,863	1.63×10^{-8}	3.72×10^{-4}	32

SNPs	EA	NEA	EAF	Beta	SE	Sample size	*P*-value	R^2	*F*-statistics
rs7206608	C	G	0.677	-0.029	0.005	55,434	1.46×10^{-8}	3.72×10^{-4}	32
rs7241572	G	A	0.791	-0.037	0.006	53,887	9.49×10^{-10}	4.42×10^{-4}	37
rs7527682	A	G	0.463	0.027	0.005	55,547	3.13×10^{-8}	3.54×10^{-4}	31
rs7541875	A	G	0.574	-0.027	0.005	55,810	1.61×10^{-8}	3.67×10^{-4}	32
rs7600261	C	T	0.694	-0.034	0.005	55,531	9.47×10^{-11}	4.86×10^{-4}	42
rs7612999	G	A	0.755	-0.031	0.006	55,396	4.90×10^{-8}	3.45×10^{-4}	30
rs761777	A	G	0.746	-0.035	0.006	55,606	4.71×10^{-10}	4.52×10^{-4}	39
rs7675588	C	A	0.205	0.034	0.006	55,455	1.80×10^{-8}	3.67×10^{-4}	32
rs7685686	A	G	0.578	0.028	0.005	55,432	1.14×10^{-8}	3.80×10^{-4}	33
rs773109	G	A	0.665	0.038	0.005	55,447	8.71×10^{-14}	6.46×10^{-4}	56
rs7942368	C	T	0.785	0.034	0.006	53,991	9.54×10^{-9}	3.89×10^{-4}	33
rs903678	G	A	0.661	-0.028	0.005	55,569	4.89×10^{-8}	3.45×10^{-4}	30
rs903959	T	A	0.601	-0.029	0.005	55,339	2.99×10^{-9}	4.08×10^{-4}	35
rs9372625	G	A	0.617	0.038	0.005	55,150	2.62×10^{-14}	6.73×10^{-4}	58
rs9373363	A	G	0.746	0.033	0.006	54,713	4.13×10^{-9}	4.04×10^{-4}	35
rs9396740	G	A	0.751	0.031	0.006	55,476	1.47×10^{-8}	3.71×10^{-4}	32
rs942065	G	A	0.366	-0.031	0.005	55,098	8.45×10^{-10}	4.38×10^{-4}	38
rs9517313	G	C	0.617	-0.033	0.005	55,658	2.05×10^{-11}	5.18×10^{-4}	45
rs9529055	G	A	0.524	-0.027	0.005	55,506	3.11×10^{-8}	3.55×10^{-4}	31
rs9542729	C	G	0.798	0.036	0.006	55,285	1.41×10^{-9}	4.26×10^{-4}	37
rs9615905	C	T	0.542	-0.028	0.005	55,538	1.21×10^{-8}	3.77×10^{-4}	32
rs9636202	G	A	0.733	0.035	0.005	55,069	1.51×10^{-10}	4.80×10^{-4}	41
rs9940128	G	A	0.578	-0.033	0.005	55,566	8.06×10^{-12}	5.39×10^{-4}	47
**63 SNPs for GRED on susceptibility (excluded confounding factors), discovery cohort**									

SNPs	EA	NEA	EAF	Beta	SE	Sample size	*P*-value	R^2	*F*-statistics
rs10133111	G	A	0.837	-0.042	0.007	55,581	1.35×10^{-10}	4.76×10^{-4}	41
rs1021363	A	G	0.358	0.031	0.005	55,401	5.10×10^{-10}	4.48×10^{-4}	39
rs10837002	C	G	0.649	-0.028	0.005	55,605	4.03×10^{-8}	3.48×10^{-4}	30
rs11762636	C	A	0.820	0.051	0.006	55,423	1.88×10^{-16}	7.83×10^{-4}	68
rs12204714	C	T	0.368	0.029	0.005	55,429	7.92×10^{-9}	3.86×10^{-4}	33
rs12453010	C	T	0.605	-0.030	0.005	55,027	1.75×10^{-9}	4.21×10^{-4}	36
rs12598916	C	G	0.725	0.033	0.005	55,368	6.87×10^{-10}	4.41×10^{-4}	38
rs12997558	G	A	0.641	-0.028	0.005	55,346	3.04×10^{-8}	3.56×10^{-4}	31
rs13107325	C	T	0.926	-0.070	0.009	55,434	2.20×10^{-14}	6.78×10^{-4}	58
rs1334297	G	A	0.266	0.039	0.005	55,300	1.14×10^{-12}	5.87×10^{-4}	51
rs13409451	A	G	0.608	0.028	0.005	55,257	1.93×10^{-8}	3.66×10^{-4}	32
rs1431196	A	G	0.572	-0.032	0.005	55,140	2.65×10^{-11}	5.15×10^{-4}	44
rs1479405	C	T	0.678	-0.031	0.005	55,492	9.85×10^{-10}	4.33×10^{-4}	37
rs1510719	T	C	0.617	0.039	0.005	55,373	3.84×10^{-15}	7.15×10^{-4}	62
rs1596747	A	G	0.506	-0.031	0.005	55,670	1.00×10^{-10}	4.83×10^{-4}	42
rs1716171	C	T	0.210	-0.038	0.006	55,381	7.82×10^{-11}	4.89×10^{-4}	42
rs17379561	A	T	0.856	-0.053	0.007	55,522	1.08×10^{-14}	6.96×10^{-4}	60
rs1883842	T	G	0.721	-0.031	0.005	55,209	9.27×10^{-9}	3.83×10^{-4}	33
rs1937450	T	G	0.462	-0.032	0.005	54,639	7.07×10^{-11}	4.96×10^{-4}	43
rs2016933	C	G	0.270	0.031	0.005	55,414	1.04×10^{-8}	3.79×10^{-4}	33
rs2023878	C	T	0.808	0.036	0.006	55,227	3.04×10^{-9}	4.09×10^{-4}	35
rs2043539	G	A	0.581	-0.027	0.005	55,631	2.24×10^{-8}	3.60×10^{-4}	31
rs2164300	C	T	0.477	0.026	0.005	55,075	4.13×10^{-8}	3.50×10^{-4}	30
rs2240326	G	A	0.526	0.047	0.005	55,674	1.13×10^{-22}	1.11×10^{-3}	96

SNPs	EA	NEA	EAF	Beta	SE	Sample size	*P*-value	R^2	*F*-statistics
rs2396133	A	G	0.525	-0.029	0.005	55,446	1.11×10^{-9}	4.30×10^{-4}	37
rs2396766	G	A	0.527	-0.032	0.005	55,425	2.33×10^{-11}	5.17×10^{-4}	45
rs2734839	C	T	0.393	0.028	0.005	55,355	8.79×10^{-9}	3.84×10^{-4}	33
rs2744961	C	T	0.642	-0.029	0.005	55,635	5.81×10^{-9}	3.92×10^{-4}	34
rs2834005	T	C	0.685	-0.030	0.005	55,500	9.42×10^{-9}	3.81×10^{-4}	33
rs2838771	G	C	0.353	0.028	0.005	55,271	2.91×10^{-8}	3.61×10^{-4}	31
rs3766823	G	A	0.829	-0.039	0.006	55,515	7.09×10^{-10}	4.40×10^{-4}	38
rs3793577	A	G	0.462	-0.027	0.005	54,460	2.49×10^{-8}	3.63×10^{-4}	31
rs3828917	G	T	0.958	-0.067	0.012	55,673	2.27×10^{-8}	3.61×10^{-4}	31
rs3863241	C	T	0.473	-0.032	0.005	55,578	1.49×10^{-11}	5.27×10^{-4}	46
rs4382592	T	G	0.300	0.030	0.005	55,446	8.20×10^{-9}	3.85×10^{-4}	33
rs4713692	C	T	0.632	0.028	0.005	55,721	3.07×10^{-8}	3.55×10^{-4}	31
rs569356	A	G	0.859	0.038	0.007	55,601	4.07×10^{-8}	3.48×10^{-4}	30
rs6722661	G	A	0.635	0.032	0.005	55,396	1.15×10^{-10}	4.82×10^{-4}	42
rs6780459	A	T	0.253	-0.031	0.006	55,576	3.14×10^{-8}	3.53×10^{-4}	31
rs7032155	C	A	0.408	-0.028	0.005	54,863	1.63×10^{-8}	3.72×10^{-4}	32
rs7206608	C	G	0.677	-0.029	0.005	55,434	1.46×10^{-8}	3.72×10^{-4}	32
rs7241572	G	A	0.791	-0.037	0.006	53,887	9.49×10^{-10}	4.42×10^{-4}	37
rs7527682	A	G	0.463	0.027	0.005	55,547	3.13×10^{-8}	3.54×10^{-4}	31
rs7541875	A	G	0.574	-0.027	0.005	55,810	1.61×10^{-8}	3.67×10^{-4}	32
rs7600261	C	T	0.694	-0.034	0.005	55,531	9.47×10^{-11}	4.86×10^{-4}	42
rs7612999	G	A	0.755	-0.031	0.006	55,396	4.90×10^{-8}	3.45×10^{-4}	30
rs761777	A	G	0.746	-0.035	0.006	55,606	4.71×10^{-10}	4.52×10^{-4}	39
rs7675588	C	A	0.205	0.034	0.006	55,455	1.80×10^{-8}	3.67×10^{-4}	32

SNPs	EA	NEA	EAF	Beta	SE	Sample size	*P*-value	R^2	*F*-statistics
rs7685686	A	G	0.578	0.028	0.005	55,432	1.14×10^{-8}	3.80×10^{-4}	33
rs773109	G	A	0.665	0.038	0.005	55,447	8.71×10^{-14}	6.46×10^{-4}	56
rs7942368	C	T	0.785	0.034	0.006	53,991	9.54×10^{-9}	3.89×10^{-4}	33
rs903678	G	A	0.661	-0.028	0.005	55,569	4.89×10^{-8}	3.45×10^{-4}	30
rs903959	T	A	0.601	-0.029	0.005	55,339	2.99×10^{-9}	4.08×10^{-4}	35
rs9372625	G	A	0.617	0.038	0.005	55,150	2.62×10^{-14}	6.73×10^{-4}	58
rs9373363	A	G	0.746	0.033	0.006	54,713	4.13×10^{-9}	4.04×10^{-4}	35
rs9396740	G	A	0.751	0.031	0.006	55,476	1.47×10^{-8}	3.71×10^{-4}	32
rs942065	G	A	0.366	-0.031	0.005	55,098	8.45×10^{-10}	4.38×10^{-4}	38
rs9517313	G	C	0.617	-0.033	0.005	55,658	2.05×10^{-11}	5.18×10^{-4}	45
rs9529055	G	A	0.524	-0.027	0.005	55,506	3.11×10^{-8}	3.55×10^{-4}	31
rs9542729	C	G	0.798	0.036	0.006	55,285	1.41×10^{-9}	4.26×10^{-4}	37
rs9615905	C	T	0.542	-0.028	0.005	55,538	1.21×10^{-8}	3.77×10^{-4}	32
rs9636202	G	A	0.733	0.035	0.005	55,069	1.51×10^{-10}	4.80×10^{-4}	41
rs9940128	G	A	0.578	-0.033	0.005	55,566	8.06×10^{-12}	5.39×10^{-4}	47
**7 SNPs for GRED on FVC, replication cohort**									
rs12238219	A	G	0.312	0.050	0.010	375,082	4.25×10^{-7}	1.08×10^{-3}	26
rs13140558	A	C	0.178	0.060	0.012	375,082	7.84×10^{-7}	1.04×10^{-3}	24
rs1549726	G	A	0.371	0.049	0.009	375,082	1.80×10^{-7}	1.14×10^{-3}	27
rs4269485	G	A	0.541	-0.049	0.009	375,082	1.10×10^{-7}	1.19×10^{-3}	28
rs4725102	C	T	0.980	0.171	0.035	375,082	8.36×10^{-7}	1.16×10^{-3}	24
rs79348626	A	G	0.017	0.179	0.033	375,082	8.79×10^{-8}	1.08×10^{-3}	299
rs7946110	G	A	0.810	0.059	0.012	375,082	5.92×10^{-7}	1.08×10^{-3}	25
**7 SNPs for GRED on DLco, replication cohort**									

SNPs	EA	NEA	EAF	Beta	SE	Sample size	*P*-value	R^2	*F*-statistics
rs12238219	A	G	0.312	0.050	0.010	375,082	4.25×10^{-7}	1.08×10^{-3}	26
rs13140558	A	C	0.178	0.060	0.012	375,082	7.84×10^{-7}	1.04×10^{-3}	24
rs1549726	G	A	0.371	0.049	0.009	375,082	1.80×10^{-7}	1.14×10^{-3}	27
rs4269485	G	A	0.541	-0.049	0.009	375,082	1.10×10^{-7}	1.19×10^{-3}	28
rs4725102	C	T	0.980	0.171	0.035	375,082	8.36×10^{-7}	1.16×10^{-3}	24
rs79348626	A	G	0.017	0.179	0.033	375,082	8.79×10^{-8}	1.08×10^{-3}	299
rs7946110	G	A	0.810	0.059	0.012	375,082	5.92×10^{-7}	1.08×10^{-3}	25
**7 SNPs for GRED on TFS, replication cohort**									
rs13140558	A	C	0.312	0.050	0.012	375,082	7.84×10^{-7}	1.04×10^{-3}	24
rs1549726	G	A	0.178	0.060	0.009	375,082	1.80×10^{-7}	1.14×10^{-3}	27
rs36110110	C	A	0.371	0.049	0.0766	375,082	3.86×10^{-7}	9.52×10^{-4}	26
rs4269485	G	A	0.541	-0.049	0.009	375,082	1.10×10^{-7}	1.19×10^{-3}	28
rs4725102	C	T	0.980	0.171	0.036	375,082	8.36×10^{-7}	1.16×10^{-3}	24
rs79348626	A	G	0.017	0.179	0.033	375,082	8.79×10^{-8}	1.08×10^{-3}	29
rs7946110	G	A	0.801	0.059	0.012	375,082	5.92×10^{-7}	1.08×10^{-3}	25
**9 SNPs for GRED on susceptibility, replication cohort**									
rs10887132	C	T	0.200	-0.059	0.012	375,082	3.95×10^{-7}	1.10×10^{-3}	26
rs12238219	A	G	0.312	0.050	0.010	375,082	4.25×10^{-7}	1.08×10^{-3}	26
rs13140558	A	C	0.178	0.060	0.012	375,082	7.84×10^{-7}	1.04×10^{-3}	24
rs1549726	G	A	0.371	0.049	0.009	375,082	1.80×10^{-7}	1.14×10^{-3}	27
rs36110110	C	A	0.003	0.384	0.076	375,082	3.86×10^{-7}	9.52×10^{-4}	26
rs4269485	G	A	0.541	-0.049	0.009	375,082	1.10×10^{-7}	1.19×10^{-3}	28
rs4725102	C	T	0.980	0.171	0.035	375,082	8.36×10^{-7}	1.16×10^{-3}	24
rs79348626	A	G	0.017	0.179	0.033	375,082	8.79×10^{-8}	1.08×10^{-3}	29

SNPs	EA	NEA	EAF	Beta	SE	Sample size	*P*-value	R^2	*F*-statistics
rs7946110	G	A	0.810	0.059	0.012	375,082	5.92×10^{-7}	1.08×10^{-3}	25

A *P*-value < 5×10^{-8} was considered genome-wide significant for discovery cohort (*P*-value < 1×10^{-6} for replication cohort). Abbreviations: SNP: single nucleotide polymorphism; MR: Mendelian randomization; EA: effect allele; NEA: baseline allele; EAF: effect allele frequency; SE: standard error; GERD: gastroesophageal reflux disease; FVC: forced vital capacity; DLco: diffuse lung capacity for carbon monoxide; TFS: transplantation-free survival.

Table S3 Summary information on outcome SNPs used as genetic instruments for the MR study

SNPs	EA	NEA	Sample size	EAF	Beta	SE	*P*-value
**62 SNPs for GRED on FVC, discovery cohort**							
rs1011407	A	G	1,048	0.9	0.0	18.2	0.987
rs10133111	G	A	1,048	0.8	-1.2	14.5	0.945
rs1021363	A	G	1,048	0.4	-2.4	10.9	0.867
rs10837002	C	G	1,048	0.7	-6.0	12.1	0.613
rs11762636	C	A	1,048	0.8	14.4	14.5	0.325
rs12204714	C	T	1,048	0.4	9.6	10.9	0.398
rs12453010	C	T	1,048	0.6	-1.2	10.9	0.936
rs12598916	C	G	1,048	0.7	20.4	12.1	0.094
rs12997558	G	A	1,048	0.7	-13.2	12.1	0.259
rs1334297	G	A	1,048	0.3	12.0	13.3	0.363
rs13409451	A	G	1,048	0.6	-1.2	10.9	0.930
rs1431196	A	G	1,048	0.6	-13.2	10.9	0.226
rs1592757	G	C	1,048	0.6	-13.2	10.9	0.247
rs1596747	A	G	1,048	0.5	8.4	10.9	0.457
rs1716171	C	T	1,048	0.2	15.6	14.5	0.244
rs17379561	A	T	1,048	0.9	8.4	16.9	0.610
rs1883842	T	G	1,048	0.7	-1.2	12.1	0.888
rs1937450	T	G	1,048	0.5	-2.4	10.9	0.817
rs2016933	C	G	1,048	0.3	36.0	13.3	0.005
rs2023878	C	T	1,048	0.8	7.2	14.5	0.616
rs2043539	G	A	1,048	0.6	3.6	10.9	0.785
rs2164300	C	T	1,048	0.5	-15.6	10.9	0.169
rs2240326	G	A	1,048	0.5	-18.0	12.1	0.117
rs2396133	A	G	1,048	0.5	3.6	10.9	0.787
rs2396766	G	A	1,048	0.5	-10.8	10.9	0.340
rs2613505	C	T	1,048	0.2	1.2	14.5	0.963
rs2734839	C	T	1,048	0.4	4.8	10.9	0.686
rs2744961	C	T	1,048	0.6	1.2	12.1	0.909
rs2834005	T	C	1,048	0.7	6.0	12.1	0.584
rs2838771	G	C	1,048	0.4	0.0	12.1	0.967
rs3766823	G	A	1,048	0.8	3.6	14.5	0.794
rs3793577	A	G	1,048	0.5	1.2	10.9	0.917
rs3828917	G	T	1,048	1.0	3.6	32.7	0.909
rs3863241	C	T	1,048	0.4	14.4	10.9	0.201
rs4382592	T	G	1,048	0.3	-12.0	12.1	0.336
rs4713692	C	T	1,048	0.6	-7.2	12.1	0.582
rs569356	A	G	1,048	0.9	8.4	16.9	0.617
rs6711584	G	A	1,048	0.6	6.0	12.1	0.611

SNPs	EA	NEA	Sample size	EAF	Beta	SE	*P*-value
rs6722661	G	A	1,048	0.6	13.2	12.1	0.267
rs6780459	A	T	1,048	0.2	2.4	13.3	0.863
rs7032155	C	A	1,048	0.4	-1.2	10.9	0.919
rs7206608	C	G	1,048	0.7	-19.2	12.1	0.119
rs7241572	G	A	1,048	0.8	7.2	14.5	0.610
rs7527682	A	G	1,048	0.5	-12.0	12.1	0.292
rs7541875	A	G	1,048	0.6	-8.4	10.9	0.454
rs7600261	C	T	1,048	0.7	-1.2	12.1	0.941
rs7612999	G	A	1,048	0.7	10.8	13.3	0.437
rs761777	A	G	1,048	0.7	-20.4	13.3	0.116
rs7675588	C	A	1,048	0.2	7.2	13.3	0.578
rs7685686	A	G	1,048	0.6	1.2	10.9	0.883
rs7942368	C	T	1,048	0.8	-31.2	13.3	0.024
rs903678	G	A	1,048	0.6	3.6	12.1	0.731
rs903959	T	A	1,048	0.6	-4.8	10.9	0.639
rs9373363	A	G	1,048	0.7	3.6	13.3	0.779
rs9396740	G	A	1,048	0.7	-2.4	13.3	0.882
rs942065	G	A	1,048	0.4	-3.6	12.1	0.736
rs9517313	G	C	1,048	0.6	-33.6	12.1	0.003
rs9529055	G	A	1,048	0.5	-10.8	12.1	0.370
rs9542729	C	G	1,048	0.8	-14.4	14.5	0.325
rs9615905	C	T	1,048	0.5	8.4	10.9	0.438
rs9636202	G	A	1,048	0.7	-4.8	12.1	0.682
rs9940128	G	A	1,048	0.6	16.8	10.9	0.137
**61 SNPs for GRED on DLco, discovery cohort**							
rs1011407	A	G	729	0.891	-0.073	0.042	0.082
rs10133111	G	A	729	0.831	-0.002	0.035	0.931
rs1021363	A	G	729	0.368	0.028	0.026	0.304
rs10837002	C	G	729	0.665	-0.047	0.028	0.089
rs11762636	C	A	729	0.831	-0.005	0.035	0.886
rs12204714	C	T	729	0.372	0.043	0.026	0.106
rs12453010	C	T	729	0.574	-0.024	0.028	0.388
rs12598916	C	G	729	0.728	0.026	0.029	0.349
rs12997558	G	A	729	0.665	0.000	0.028	0.992
rs1334297	G	A	729	0.262	0.052	0.031	0.093
rs13409451	A	G	654	0.596	0.058	0.028	0.037
rs1431196	A	G	729	0.561	-0.061	0.028	0.031
rs1592757	G	C	729	0.626	-0.036	0.026	0.172
rs1596747	A	G	729	0.511	0.036	0.025	0.164
rs1716171	C	T	729	0.189	0.030	0.034	0.362
rs17379561	A	T	654	0.856	-0.006	0.042	0.876
rs1883842	T	G	729	0.722	0.016	0.029	0.583

SNPs	EA	NEA	Sample size	EAF	Beta	SE	*P*-value
rs1937450	T	G	729	0.457	-0.031	0.028	0.251
rs2016933	C	G	654	0.277	0.043	0.032	0.187
rs2023878	C	T	729	0.828	0.020	0.036	0.563
rs2043539	G	A	729	0.590	0.019	0.026	0.477
rs2164300	C	T	729	0.469	-0.038	0.026	0.142
rs2240326	G	A	729	0.532	-0.022	0.026	0.406
rs2396133	A	G	729	0.494	-0.026	0.023	0.261
rs2396766	G	A	729	0.523	0.017	0.026	0.535
rs2613505	C	T	729	0.192	-0.038	0.034	0.250
rs2734839	C	T	729	0.388	0.050	0.028	0.062
rs2744961	C	T	729	0.614	0.038	0.028	0.157
rs2834005	T	C	729	0.703	-0.032	0.028	0.256
rs2838771	G	C	729	0.363	0.031	0.028	0.273
rs3766823	G	A	729	0.821	-0.004	0.035	0.926
rs3793577	A	G	729	0.473	-0.018	0.023	0.434
rs3828917	G	T	729	0.970	-0.030	0.082	0.716
rs3863241	C	T	729	0.452	-0.001	0.025	0.981
rs4382592	T	G	729	0.295	-0.041	0.029	0.150
rs4713692	C	T	729	0.642	-0.043	0.030	0.148
rs569356	A	G	729	0.875	0.025	0.041	0.524
rs6711584	G	A	729	0.546	0.004	0.028	0.904
rs6722661	G	A	729	0.632	-0.012	0.029	0.684
rs6780459	A	T	729	0.227	0.010	0.030	0.750
rs7032155	C	A	729	0.404	0.013	0.026	0.613
rs7206608	C	G	729	0.671	0.000	0.030	0.998
rs7241572	G	A	729	0.805	-0.004	0.035	0.927
rs7527682	A	G	729	0.472	0.017	0.028	0.526
rs7541875	A	G	729	0.584	0.006	0.026	0.822
rs7600261	C	T	729	0.705	-0.028	0.029	0.341
rs7612999	G	A	729	0.749	0.029	0.031	0.367
rs761777	A	G	729	0.729	-0.012	0.030	0.681
rs7675588	C	A	654	0.224	0.008	0.034	0.789
rs7685686	A	G	729	0.559	-0.022	0.026	0.421
rs7942368	C	T	654	0.779	-0.036	0.035	0.308
rs903678	G	A	654	0.646	-0.032	0.029	0.259
rs903959	T	A	729	0.609	0.036	0.026	0.170
rs9373363	A	G	654	0.746	0.016	0.031	0.610
rs9396740	G	A	729	0.762	0.036	0.031	0.249
rs942065	G	A	729	0.357	0.013	0.026	0.638
rs9529055	G	A	729	0.522	0.061	0.028	0.026
rs9542729	C	G	729	0.829	-0.029	0.035	0.401
rs9615905	C	T	654	0.543	0.016	0.029	0.580

SNPs	EA	NEA	Sample size	EAF	Beta	SE	*P*-value
rs9636202	G	A	729	0.729	0.040	0.030	0.188
rs9940128	G	A	729	0.574	0.023	0.026	0.403
**60 SNPs for GRED on TFS, discovery cohort**							
rs1011407	A	G	1,481	0.895	0.066	0.081	0.413
rs10133111	G	A	1,481	0.823	-0.096	0.066	0.145
rs1021363	A	G	1,481	0.378	0.044	0.052	0.398
rs10837002	C	G	1,481	0.663	-0.013	0.052	0.800
rs11762636	C	A	1,481	0.823	-0.098	0.065	0.134
rs11953061	C	T	1,481	0.677	-0.001	0.052	0.992
rs12204714	C	T	1,481	0.365	-0.010	0.052	0.853
rs12453010	C	T	1,481	0.584	-0.014	0.052	0.782
rs12598916	C	G	1,481	0.713	-0.001	0.057	0.984
rs12997558	G	A	1,481	0.641	0.045	0.054	0.410
rs1334297	G	A	1,481	0.261	-0.127	0.060	0.035
rs13409451	A	G	1,481	0.610	0.041	0.053	0.441
rs1431196	A	G	1,481	0.577	0.010	0.052	0.848
rs1592757	G	C	1,481	0.642	0.009	0.053	0.872
rs1596747	A	G	1,481	0.489	0.023	0.050	0.647
rs1716171	C	T	1,481	0.191	-0.068	0.066	0.306
rs17379561	A	T	1,481	0.867	0.044	0.070	0.532
rs1883842	T	G	1,481	0.725	0.090	0.058	0.120
rs1937450	T	G	1,481	0.427	-0.044	0.052	0.396
rs2016933	C	G	1,481	0.275	0.114	0.059	0.052
rs2023878	C	T	1,481	0.818	-0.048	0.065	0.463
rs2043539	G	A	1,481	0.593	0.014	0.051	0.780
rs2106353	G	T	1,481	0.770	0.043	0.062	0.483
rs2164300	C	T	1,481	0.442	-0.005	0.052	0.930
rs2240326	G	A	1,481	0.521	0.045	0.051	0.377
rs2396133	A	G	1,481	0.520	0.039	0.050	0.436
rs2396766	G	A	1,481	0.527	-0.024	0.051	0.639
rs2613505	C	T	1,481	0.188	0.013	0.066	0.846
rs2734839	C	T	1,481	0.381	-0.039	0.052	0.451
rs2744961	C	T	1,481	0.610	0.050	0.053	0.347
rs2834005	T	C	1,481	0.699	-0.031	0.056	0.575
rs2838771	G	C	1,481	0.374	-0.009	0.054	0.864
rs3766823	G	A	1,481	0.799	-0.057	0.065	0.384
rs3828917	G	T	1,481	0.965	-0.342	0.124	0.006
rs3863241	C	T	1,481	0.479	0.055	0.051	0.287
rs4713692	C	T	1,481	0.622	0.032	0.054	0.556
rs569356	A	G	1,481	0.869	0.043	0.072	0.555
rs6711584	G	A	1,481	0.522	-0.077	0.052	0.139
rs6722661	G	A	1,481	0.658	-0.015	0.053	0.783

SNPs	EA	NEA	Sample size	EAF	Beta	SE	*P*-value
rs6780459	A	T	1,481	0.216	-0.064	0.058	0.275
rs7206608	C	G	1,481	0.673	-0.011	0.055	0.847
rs7241572	G	A	1,481	0.793	0.046	0.065	0.478
rs7527682	A	G	1,481	0.440	-0.006	0.051	0.901
rs7541875	A	G	1,481	0.577	0.035	0.051	0.495
rs7600261	C	T	1,481	0.690	0.067	0.055	0.226
rs7612999	G	A	1,481	0.738	0.021	0.058	0.717
rs761777	A	G	1,481	0.748	-0.096	0.061	0.116
rs7675588	C	A	1,481	0.209	-0.048	0.061	0.429
rs7685686	A	G	1,481	0.565	-0.016	0.051	0.753
rs7942368	C	T	1,481	0.805	0.023	0.061	0.711
rs903678	G	A	1,481	0.643	0.047	0.055	0.396
rs9373363	A	G	1,481	0.735	-0.050	0.059	0.390
rs9396740	G	A	1,481	0.774	-0.066	0.060	0.273
rs942065	G	A	1,481	0.355	0.048	0.054	0.368
rs9517313	G	C	1,481	0.633	0.024	0.053	0.650
rs9529055	G	A	1,481	0.532	0.018	0.052	0.723
rs9542729	C	G	1,481	0.822	0.062	0.063	0.325
rs9615905	C	T	1,481	0.551	0.080	0.054	0.140
rs9636202	G	A	1,481	0.715	-0.081	0.060	0.176
rs9940128	G	A	1,481	0.580	0.006	0.052	0.908
**75 SNPs for GRED on susceptibility, discovery cohort**							
rs10010963	C	T	24,589	0.385	0.020	0.028	0.470
rs1011407	A	G	24,589	0.881	0.145	0.043	0.001
rs10133111	G	A	24,589	0.169	-0.080	0.035	0.024
rs1021363	A	G	22,051	0.643	0.016	0.031	0.605
rs10837002	C	G	24,589	0.647	-0.006	0.029	0.847
rs11762636	C	A	24,589	0.188	0.004	0.034	0.912
rs12204714	C	T	22,051	0.360	0.002	0.031	0.959
rs12357321	G	A	24,589	0.322	0.009	0.029	0.759
rs12453010	C	T	22,051	0.397	-0.048	0.030	0.116
rs12598916	C	G	24,589	0.723	0.000	0.030	0.990
rs12967855	A	G	24,589	0.671	0.042	0.029	0.146
rs12997558	G	A	24,589	0.355	0.014	0.028	0.621
rs13107325	C	T	24,589	0.075	-0.011	0.051	0.838
rs1334297	G	A	24,589	0.262	0.020	0.031	0.517
rs13409451	A	G	22,051	0.613	-0.022	0.030	0.475
rs1431196	A	G	24,589	0.569	0.002	0.027	0.930
rs1479405	C	T	24,589	0.323	-0.047	0.029	0.106
rs1510719	T	C	24,589	0.621	-0.008	0.028	0.776
rs1592757	G	C	24,589	0.642	0.036	0.028	0.205
rs1596747	A	G	24,589	0.509	0.000	0.027	0.988

SNPs	EA	NEA	Sample size	EAF	Beta	SE	*P*-value
rs1716171	C	T	24,589	0.210	-0.071	0.033	0.032
rs17379561	A	T	24,589	0.857	-0.091	0.038	0.019
rs1883842	T	G	24,589	0.726	-0.012	0.030	0.686
rs1937450	T	G	24,589	0.534	-0.060	0.027	0.030
rs2016933	C	G	24,589	0.277	-0.049	0.030	0.101
rs2023878	C	T	24,589	0.190	0.023	0.035	0.502
rs2043539	G	A	24,589	0.418	0.012	0.028	0.669
rs215614	G	A	24,589	0.373	0.017	0.028	0.549
rs2164300	C	T	24,589	0.484	-0.052	0.027	0.058
rs2240326	G	A	24,589	0.483	0.022	0.027	0.425
rs2396133	A	G	24,589	0.527	-0.041	0.027	0.131
rs2396766	G	A	24,589	0.462	0.012	0.027	0.672
rs2613505	C	T	24,589	0.187	-0.029	0.035	0.413
rs2734839	C	T	24,589	0.389	-0.003	0.028	0.917
rs2744961	C	T	24,589	0.368	-0.068	0.028	0.015
rs2782641	G	A	24,589	0.385	-0.045	0.028	0.108
rs2834005	T	C	22,051	0.686	0.006	0.032	0.847
rs2838771	G	C	24,589	0.349	-0.019	0.029	0.511
rs324769	C	T	24,589	0.446	-0.001	0.028	0.965
rs329122	G	A	24,589	0.418	0.004	0.028	0.896
rs3766823	G	A	24,589	0.176	0.028	0.036	0.437
rs3793577	A	G	24,589	0.535	0.010	0.027	0.726
rs3828917	G	T	22,051	0.040	0.046	0.077	0.550
rs3863241	C	T	24,589	0.462	-0.037	0.027	0.173
rs4300861	C	T	24,589	0.379	-0.041	0.028	0.141
rs4382592	T	G	24,589	0.694	-0.030	0.029	0.299
rs4713692	C	T	24,589	0.358	-0.029	0.028	0.300
rs569356	A	G	24,589	0.859	0.034	0.039	0.385
rs6711584	G	A	24,589	0.457	0.030	0.028	0.271
rs6722661	G	A	24,589	0.358	0.048	0.028	0.088
rs6780459	A	T	24,589	0.255	0.043	0.031	0.164
rs7032155	C	A	24,589	0.401	-0.009	0.028	0.741
rs7206608	C	G	24,589	0.679	-0.028	0.029	0.332
rs7241572	G	A	24,589	0.202	0.004	0.034	0.916
rs7527682	A	G	24,589	0.538	-0.030	0.027	0.273
rs7541875	A	G	24,589	0.579	-0.024	0.028	0.393
rs7600261	C	T	24,589	0.302	0.023	0.030	0.437
rs7612999	G	A	24,589	0.249	-0.023	0.031	0.468
rs761777	A	G	24,589	0.740	-0.008	0.031	0.788
rs7675588	C	A	24,589	0.207	0.022	0.033	0.517
rs7685686	A	G	24,589	0.577	-0.057	0.028	0.037
rs773109	G	A	24,589	0.329	0.015	0.029	0.615

SNPs	EA	NEA	Sample size	EAF	Beta	SE	*P*-value
rs7942368	C	T	24,589	0.219	-0.020	0.033	0.548
rs903678	G	A	24,589	0.335	-0.010	0.029	0.737
rs903959	T	A	24,589	0.604	0.003	0.028	0.923
rs9372625	G	A	24,589	0.381	-0.023	0.028	0.404
rs9373363	A	G	24,589	0.754	-0.044	0.032	0.161
rs9396740	G	A	24,589	0.252	-0.032	0.031	0.297
rs942065	G	A	22,051	0.364	-0.057	0.031	0.064
rs9517313	G	C	22,051	0.620	-0.026	0.030	0.401
rs9529055	G	A	24,589	0.482	0.008	0.027	0.780
rs9542729	C	G	22,051	0.800	0.079	0.037	0.034
rs9615905	C	T	24,589	0.452	0.008	0.027	0.780
rs9636202	G	A	24,589	0.262	-0.005	0.031	0.875
rs9940128	G	A	24,589	0.428	0.019	0.027	0.495
**63 SNPs for GRED on susceptibility (excluded confounding factors), discovery cohort**							
rs10133111	G	A	24,589	0.169	-0.080	0.035	0.024
rs1021363	A	G	22,051	0.643	0.016	0.031	0.605
rs10837002	C	G	24,589	0.647	-0.006	0.029	0.847
rs11762636	C	A	24,589	0.188	0.004	0.034	0.912
rs12204714	C	T	22,051	0.360	0.002	0.031	0.959
rs12453010	C	T	22,051	0.397	-0.048	0.030	0.116
rs12598916	C	G	24,589	0.723	0.000	0.030	0.990
rs12997558	G	A	24,589	0.355	0.014	0.028	0.621
rs13107325	C	T	24,589	0.075	-0.011	0.051	0.838
rs1334297	G	A	24,589	0.262	0.020	0.031	0.517
rs13409451	A	G	22,051	0.613	-0.022	0.030	0.475
rs1431196	A	G	24,589	0.569	0.002	0.027	0.930
rs1479405	C	T	24,589	0.323	-0.047	0.029	0.106
rs1510719	T	C	24,589	0.621	-0.008	0.028	0.776
rs1596747	A	G	24,589	0.509	0.000	0.027	0.988
rs1716171	C	T	24,589	0.210	-0.071	0.033	0.032
rs17379561	A	T	24,589	0.857	-0.091	0.038	0.019
rs1883842	T	G	24,589	0.726	-0.012	0.030	0.686
rs1937450	T	G	24,589	0.534	-0.060	0.027	0.030
rs2016933	C	G	24,589	0.277	-0.049	0.030	0.101
rs2023878	C	T	24,589	0.190	0.023	0.035	0.502
rs2043539	G	A	24,589	0.418	0.012	0.028	0.669
rs2164300	C	T	24,589	0.484	-0.052	0.027	0.058
rs2240326	G	A	24,589	0.483	0.022	0.027	0.425
rs2396133	A	G	24,589	0.527	-0.041	0.027	0.131
rs2396766	G	A	24,589	0.462	0.012	0.027	0.672

SNPs	EA	NEA	Sample size	EAF	Beta	SE	*P*-value
rs2734839	C	T	24,589	0.389	-0.003	0.028	0.917
rs2744961	C	T	24,589	0.368	-0.068	0.028	0.015
rs2834005	T	C	22,051	0.686	0.006	0.032	0.847
rs2838771	G	C	24,589	0.349	-0.019	0.029	0.511
rs3766823	G	A	24,589	0.176	0.028	0.036	0.437
rs3793577	A	G	24,589	0.535	0.010	0.027	0.726
rs3828917	G	T	22,051	0.040	0.046	0.077	0.550
rs3863241	C	T	24,589	0.462	-0.037	0.027	0.173
rs4382592	T	G	24,589	0.694	-0.030	0.029	0.299
rs4713692	C	T	24,589	0.358	-0.029	0.028	0.300
rs569356	A	G	24,589	0.859	0.034	0.039	0.385
rs6722661	G	A	24,589	0.358	0.048	0.028	0.088
rs6780459	A	T	24,589	0.255	0.043	0.031	0.164
rs7032155	C	A	24,589	0.401	-0.009	0.028	0.741
rs7206608	C	G	24,589	0.679	-0.028	0.029	0.332
rs7241572	G	A	24,589	0.202	0.004	0.034	0.916
rs7527682	A	G	24,589	0.538	-0.030	0.027	0.273
rs7541875	A	G	24,589	0.579	-0.024	0.028	0.393
rs7600261	C	T	24,589	0.302	0.023	0.030	0.437
rs7612999	G	A	24,589	0.249	-0.023	0.031	0.468
rs761777	A	G	24,589	0.740	-0.008	0.031	0.788
rs7675588	C	A	24,589	0.207	0.022	0.033	0.517
rs7685686	A	G	24,589	0.577	-0.057	0.028	0.037
rs773109	G	A	24,589	0.329	0.015	0.029	0.615
rs7942368	C	T	24,589	0.219	-0.020	0.033	0.548
rs903678	G	A	24,589	0.335	-0.010	0.029	0.737
rs903959	T	A	24,589	0.604	0.003	0.028	0.923
rs9372625	G	A	24,589	0.381	-0.023	0.028	0.404
rs9373363	A	G	24,589	0.754	-0.044	0.032	0.161
rs9396740	G	A	24,589	0.252	-0.032	0.031	0.297
rs942065	G	A	22,051	0.364	-0.057	0.031	0.064
rs9517313	G	C	22,051	0.620	-0.026	0.030	0.401
rs9529055	G	A	24,589	0.482	0.008	0.027	0.780
rs9542729	C	G	22,051	0.800	0.079	0.037	0.034
rs9615905	C	T	24,589	0.452	0.008	0.027	0.780
rs9636202	G	A	24,589	0.262	-0.005	0.031	0.875
rs9940128	G	A	24,589	0.428	0.019	0.027	0.495
**7 SNPs for GRED on FVC, replication cohort**							
rs12238219	A	G	1,048	0.245	-2.4	13.31	0.846
rs13140558	A	C	1,048	0.229	-7.2	13.31	0.579
rs1549726	G	A	1,048	0.373	2.4	12.1	0.827
rs4269485	G	A	1,048	0.692	-3.6	12.1	0.722

SNPs	EA	NEA	Sample size	EAF	Beta	SE	*P*-value
rs4725102	C	T	906	0.979	-2.4	47.19	0.962
rs79348626	A	G	1,048	0.023	-22.8	37.51	0.554
rs7946110	G	A	1,048	0.836	-18	15.73	0.250
**7 SNPs for GRED on DLco, replication cohort**							
rs12238219	A	G	729	0.249	0.043	0.032	0.178
rs13140558	A	C	729	0.232	0.037	0.032	0.250
rs1549726	G	A	729	0.378	0.006	0.028	0.837
rs4269485	G	A	729	0.704	-0.041	0.028	0.147
rs4725102	C	T	654	0.977	-0.078	0.104	0.453
rs79348626	A	G	654	0.025	0.101	0.100	0.313
rs7946110	G	A	729	0.840	-0.036	0.037	0.328
**7 SNPs for GRED on TFS, replication cohort**							
rs13140558	A	C	1,481	0.245	0.007	0.063	0.915
rs1549726	G	A	1,481	0.391	-0.014	0.052	0.790
rs36110110	C	A	1,481	0.012	-0.141	0.278	0.612
rs4269485	G	A	1,481	0.711	0.037	0.054	0.495
rs4725102	C	T	1,481	0.982	0.034	0.238	0.887
rs79348626	A	G	1,481	0.019	0.070	0.171	0.683
rs7946110	G	A	1,481	0.839	0.039	0.071	0.584
**9 SNPs for GRED on susceptibility, replication cohort**							
rs10887132	C	T	346,571	0.200	0.073	0.040	0.068
rs12238219	A	G	346,571	0.312	-0.053	0.034	0.126
rs13140558	A	C	346,571	0.178	-0.006	0.042	0.894
rs1549726	G	A	346,571	0.372	0.002	0.033	0.960
rs36110110	C	A	346,571	0.003	-0.395	0.309	0.201
rs4269485	G	A	346,571	0.540	-0.038	0.032	0.236
rs4725102	C	T	346,571	0.980	-0.080	0.114	0.485
rs79348626	A	G	346,571	0.017	-0.091	0.124	0.462
rs7946110	G	A	346,571	0.810	-0.058	0.041	0.150

A *P*-value $< 5 \times 10^{-6}$ was considered genome-wide significant and should be excluded to meet the assumption that requires instruments to be associated with the outcome only through exposure. Abbreviations: SNP: single nucleotide polymorphism; MR: Mendelian randomization; EA: effect allele; NEA: baseline allele; EAF: effect allele frequency; SE: standard error; GERD: gastroesophageal reflux disease; FVC: forced vital capacity; DLco: diffuse lung capacity for carbon monoxide; TFS: transplantation-free survival.

Table S4 The heterogeneity and pleiotropy analysis based on three different statistical methods

Exposure	Outcomes	Cochran's Q statistic				MR-PRESSO	MR-Egger intercept analysis
		MR-Egger		IVW			
		Q	*P*-value	Q	*P*-value	*P*-value	*P*-value
GERD (discovery cohort)	FVC	56.033	0.621	56.262	0.648	0.645	0.634
	DLCO	68.084	0.196	68.145	0.220	0.228	0.818
	TFS	50.860	0.736	52.876	0.699	0.710	0.161
	Susceptibility	89.195	0.096	93.889	0.0592	0.054	0.054
	Susceptibility (excluded confounding factors)	70.084	0.199	73.762	0.146	0.123	0.079
GERD (replication cohort)	FVC	1.412	0.923	1.521	0.958	0.963	0.755
	DLCO	5.386	0.371	5.953	0.428	0.470	0.501
	TFS	1.251	0.940	1.259	0.974	0.972	0.931
	Susceptibility	6.877	0.442	7.533	0.480	0.503	0.444

Abbreviations: MR: Mendelian randomization; IVW: inverse variance weighted; GERD: gastroesophageal reflux disease; FVC: forced vital capacity; DLco: diffuse lung capacity for carbon monoxide; TFS: transplantation-free survival.

Table S5 Index instrumental SNPs for exposures and outcomes in the multivariable Mendelian randomization analysis

SNPs	GERD			Smoking Initiation			IPF		
	Beta	SE	*P*-value	Beta	SE	*P*-value	Beta	SE	*P*-value
**Discovery Cohort (number of SNPs: 105)**									
rs10010963	2.70×10^{-2}	4.95×10^{-3}	4.92×10^{-8}	-2.45×10^{-3}	3.66×10^{-3}	5.04×10^{-1}	2.02×10^{-2}	2.79×10^{-2}	4.70×10^{-1}
rs10036561	-1.65×10^{-2}	5.51×10^{-3}	2.72×10^{-3}	2.35×10^{-2}	4.08×10^{-3}	9.36×10^{-9}	2.75×10^{-2}	3.13×10^{-2}	3.80×10^{-1}
rs10119773	-2.69×10^{-2}	4.86×10^{-3}	3.26×10^{-8}	1.19×10^{-2}	3.56×10^{-3}	8.30×10^{-4}	-1.10×10^{-2}	2.71×10^{-2}	6.85×10^{-1}
rs1013030	-2.96×10^{-2}	5.33×10^{-3}	2.83×10^{-8}	8.56×10^{-4}	3.97×10^{-3}	8.29×10^{-1}	-6.00×10^{-3}	3.02×10^{-2}	8.43×10^{-1}
rs10228350	-2.89×10^{-2}	4.89×10^{-3}	3.48×10^{-9}	9.80×10^{-3}	3.60×10^{-3}	6.53×10^{-3}	2.38×10^{-2}	2.77×10^{-2}	3.91×10^{-1}
rs10490159	-8.91×10^{-3}	4.91×10^{-3}	6.95×10^{-2}	2.16×10^{-2}	3.63×10^{-3}	2.59×10^{-9}	-2.31×10^{-2}	2.80×10^{-2}	4.10×10^{-1}
rs10492861	-2.98×10^{-2}	5.33×10^{-3}	2.37×10^{-8}	-4.58×10^{-3}	3.91×10^{-3}	2.41×10^{-1}	-2.83×10^{-2}	3.02×10^{-2}	3.49×10^{-1}
rs1050863	9.02×10^{-3}	4.88×10^{-3}	6.45×10^{-2}	-2.01×10^{-2}	3.58×10^{-3}	2.15×10^{-8}	-2.54×10^{-2}	2.74×10^{-2}	3.54×10^{-1}
rs10747488	4.20×10^{-2}	5.72×10^{-3}	2.27×10^{-13}	-7.91×10^{-5}	4.16×10^{-3}	9.85×10^{-1}	8.81×10^{-2}	3.21×10^{-2}	6.13×10^{-3}
rs10818398	-2.71×10^{-2}	4.91×10^{-3}	3.49×10^{-8}	-1.36×10^{-3}	3.63×10^{-3}	7.08×10^{-1}	-1.60×10^{-3}	2.77×10^{-2}	9.54×10^{-1}
rs10837002	-2.76×10^{-2}	5.04×10^{-3}	4.03×10^{-8}	5.19×10^{-3}	3.73×10^{-3}	1.64×10^{-1}	5.50×10^{-3}	2.86×10^{-2}	8.47×10^{-1}
rs11030380	-1.83×10^{-2}	4.92×10^{-3}	1.98×10^{-4}	2.01×10^{-2}	3.63×10^{-3}	2.97×10^{-8}	-8.20×10^{-3}	2.79×10^{-2}	7.69×10^{-1}
rs11057005	1.67×10^{-2}	4.87×10^{-3}	6.11×10^{-4}	-2.09×10^{-2}	3.58×10^{-3}	4.85×10^{-9}	4.63×10^{-2}	2.73×10^{-2}	8.98×10^{-2}
rs11645288	-3.39×10^{-2}	6.11×10^{-3}	2.78×10^{-8}	6.08×10^{-3}	4.50×10^{-3}	1.77×10^{-1}	-2.36×10^{-2}	3.38×10^{-2}	4.85×10^{-1}
rs11716779	-2.00×10^{-2}	5.42×10^{-3}	2.18×10^{-4}	2.20×10^{-2}	4.03×10^{-3}	4.88×10^{-8}	-3.48×10^{-2}	3.10×10^{-2}	2.61×10^{-1}
rs11780471	1.27×10^{-2}	9.91×10^{-3}	2.00×10^{-1}	-5.05×10^{-2}	7.33×10^{-3}	5.69×10^{-12}	1.68×10^{-2}	5.58×10^{-2}	7.63×10^{-1}
rs11992983	-3.10×10^{-2}	4.85×10^{-3}	1.64×10^{-10}	1.14×10^{-2}	3.57×10^{-3}	1.44×10^{-3}	-4.17×10^{-2}	2.74×10^{-2}	1.28×10^{-1}
rs12442894	-1.09×10^{-2}	5.82×10^{-3}	6.20×10^{-2}	-2.44×10^{-2}	4.29×10^{-3}	1.28×10^{-8}	9.00×10^{-3}	3.30×10^{-2}	7.85×10^{-1}
rs1261097	-3.12×10^{-2}	5.26×10^{-3}	2.96×10^{-9}	-5.88×10^{-3}	3.89×10^{-3}	1.31×10^{-1}	5.00×10^{-4}	2.98×10^{-2}	9.87×10^{-1}
rs12617870	-2.67×10^{-2}	4.84×10^{-3}	3.67×10^{-8}	8.15×10^{-4}	3.57×10^{-3}	8.19×10^{-1}	-7.10×10^{-3}	2.71×10^{-2}	7.93×10^{-1}
rs12662100	3.24×10^{-2}	5.55×10^{-3}	5.22×10^{-9}	-1.50×10^{-3}	4.12×10^{-3}	7.15×10^{-1}	1.07×10^{-2}	3.07×10^{-2}	7.27×10^{-1}

SNPs	GERD			Smoking Initiation			IPF		
	**Beta**	**SE**	***P*-value**	**Beta**	**SE**	***P*-value**	**Beta**	**SE**	***P*-value**
rs12662631	-1.17×10^{-2}	5.18×10^{-3}	2.39×10^{-2}	2.10×10^{-2}	3.83×10^{-3}	4.26×10^{-8}	8.54×10^{-2}	2.92×10^{-2}	3.44×10^{-3}
rs12888955	2.85×10^{-2}	5.07×10^{-3}	1.75×10^{-8}	-9.27×10^{-3}	3.74×10^{-3}	1.32×10^{-2}	4.26×10^{-2}	2.83×10^{-2}	1.33×10^{-1}
rs12939066	-3.02×10^{-2}	5.11×10^{-3}	3.13×10^{-9}	1.64×10^{-2}	3.80×10^{-3}	1.59×10^{-5}	-6.49×10^{-2}	2.89×10^{-2}	2.46×10^{-2}
rs12966968	-2.95×10^{-2}	4.90×10^{-3}	1.62×10^{-9}	7.74×10^{-3}	3.61×10^{-3}	3.22×10^{-2}	1.43×10^{-2}	2.74×10^{-2}	6.02×10^{-1}
rs12985909	3.05×10^{-2}	4.84×10^{-3}	2.81×10^{-10}	-1.13×10^{-2}	3.58×10^{-3}	1.57×10^{-3}	4.50×10^{-3}	2.73×10^{-2}	8.69×10^{-1}
rs12997558	-2.78×10^{-2}	5.02×10^{-3}	3.04×10^{-8}	2.38×10^{-3}	3.70×10^{-3}	5.20×10^{-1}	1.39×10^{-2}	2.81×10^{-2}	6.21×10^{-1}
rs13019618	3.09×10^{-2}	5.67×10^{-3}	4.86×10^{-8}	4.64×10^{-3}	4.21×10^{-3}	2.70×10^{-1}	7.59×10^{-2}	3.18×10^{-2}	1.71×10^{-2}
rs13227117	2.67×10^{-2}	4.89×10^{-3}	4.86×10^{-8}	-8.97×10^{-3}	3.59×10^{-3}	1.25×10^{-2}	1.00×10^{-3}	2.75×10^{-2}	9.71×10^{-1}
rs13409451	2.77×10^{-2}	4.93×10^{-3}	1.93×10^{-8}	-5.55×10^{-3}	3.65×10^{-3}	1.28×10^{-1}	2.17×10^{-2}	3.04×10^{-2}	4.75×10^{-1}
rs1343424	-1.98×10^{-2}	5.54×10^{-3}	3.49×10^{-4}	2.28×10^{-2}	4.17×10^{-3}	4.67×10^{-8}	2.04×10^{-2}	3.17×10^{-2}	5.20×10^{-1}
rs134529	4.70×10^{-3}	4.95×10^{-3}	3.42×10^{-1}	-2.00×10^{-2}	3.66×10^{-3}	4.85×10^{-8}	4.92×10^{-2}	2.81×10^{-2}	8.04×10^{-2}
rs1346622	2.89×10^{-2}	4.88×10^{-3}	2.94×10^{-9}	3.30×10^{-3}	3.77×10^{-3}	3.82×10^{-1}	1.19×10^{-2}	2.74×10^{-2}	6.64×10^{-1}
rs1427499	5.51×10^{-3}	5.31×10^{-3}	2.99×10^{-1}	-2.24×10^{-2}	3.92×10^{-3}	1.08×10^{-8}	5.16×10^{-2}	3.01×10^{-2}	8.69×10^{-2}
rs1436351	-3.05×10^{-2}	5.52×10^{-3}	3.14×10^{-8}	3.51×10^{-3}	4.11×10^{-3}	3.93×10^{-1}	-4.50×10^{-2}	3.12×10^{-2}	1.49×10^{-1}
rs1466802	-1.39×10^{-2}	5.77×10^{-3}	1.60×10^{-2}	2.35×10^{-2}	4.21×10^{-3}	2.21×10^{-8}	3.45×10^{-2}	3.24×10^{-2}	2.86×10^{-1}
rs1486465	-1.31×10^{-2}	5.57×10^{-3}	1.86×10^{-2}	2.25×10^{-2}	4.10×10^{-3}	3.94×10^{-8}	-2.10×10^{-3}	3.12×10^{-2}	9.46×10^{-1}
rs1570059	-3.33×10^{-2}	4.82×10^{-3}	4.79×10^{-12}	1.25×10^{-2}	3.57×10^{-3}	4.56×10^{-4}	4.46×10^{-2}	2.73×10^{-2}	1.02×10^{-1}
rs17613129	-9.92×10^{-3}	5.25×10^{-3}	5.89×10^{-2}	2.19×10^{-2}	3.90×10^{-3}	1.89×10^{-8}	2.50×10^{-3}	2.96×10^{-2}	9.33×10^{-1}
rs17701934	2.65×10^{-2}	4.85×10^{-3}	4.60×10^{-8}	2.22×10^{-3}	3.60×10^{-3}	5.38×10^{-1}	-8.00×10^{-4}	2.75×10^{-2}	9.77×10^{-1}
rs17822102	2.95×10^{-2}	5.06×10^{-3}	5.24×10^{-9}	-8.26×10^{-3}	3.74×10^{-3}	2.74×10^{-2}	-3.50×10^{-3}	2.88×10^{-2}	9.03×10^{-1}
rs1860224	2.94×10^{-2}	5.35×10^{-3}	3.70×10^{-8}	-6.15×10^{-3}	3.92×10^{-3}	1.16×10^{-1}	-6.80×10^{-3}	3.01×10^{-2}	8.22×10^{-1}
rs199752	3.04×10^{-2}	5.31×10^{-3}	1.01×10^{-8}	-7.80×10^{-3}	3.90×10^{-3}	4.58×10^{-2}	2.08×10^{-2}	2.95×10^{-2}	4.81×10^{-1}

SNPs		GERD			Smoking Initiation			IPF	
	**Beta**	**SE**	***P*-value**	**Beta**	**SE**	***P*-value**	**Beta**	**SE**	***P*-value**
rs2046850	1.29×10^{-2}	6.08×10^{-3}	3.41×10^{-2}	-2.48×10^{-2}	4.48×10^{-3}	3.03×10^{-8}	3.62×10^{-2}	3.49×10^{-2}	2.99×10^{-1}
rs205319	-2.75×10^{-2}	4.87×10^{-3}	1.73×10^{-8}	8.65×10^{-3}	3.60×10^{-3}	1.63×10^{-2}	5.88×10^{-2}	3.00×10^{-2}	5.03×10^{-2}
rs2069408	3.03×10^{-2}	5.08×10^{-3}	2.28×10^{-9}	-1.01×10^{-2}	3.78×10^{-3}	7.26×10^{-3}	6.10×10^{-3}	2.88×10^{-2}	8.32×10^{-1}
rs2107300	2.71×10^{-2}	6.63×10^{-3}	4.45×10^{-5}	-2.72×10^{-2}	4.93×10^{-3}	3.27×10^{-8}	-1.10×10^{-2}	3.75×10^{-2}	7.69×10^{-1}
rs2111705	-2.77×10^{-2}	4.83×10^{-3}	9.02×10^{-9}	4.70×10^{-3}	3.57×10^{-3}	1.88×10^{-1}	-3.17×10^{-2}	2.72×10^{-2}	2.44×10^{-1}
rs2240294	1.11×10^{-2}	4.85×10^{-3}	2.18×10^{-2}	-2.09×10^{-2}	3.57×10^{-3}	4.82×10^{-9}	-4.00×10^{-3}	2.98×10^{-2}	8.93×10^{-1}
rs2281767	-2.66×10^{-2}	4.83×10^{-3}	3.81×10^{-8}	2.71×10^{-3}	3.56×10^{-3}	4.45×10^{-1}	1.03×10^{-2}	2.72×10^{-2}	7.05×10^{-1}
rs2285086	2.67×10^{-2}	4.89×10^{-3}	4.86×10^{-8}	-8.95×10^{-4}	3.59×10^{-3}	8.03×10^{-1}	4.07×10^{-2}	2.75×10^{-2}	1.39×10^{-1}
rs2397135	-5.65×10^{-3}	5.47×10^{-3}	3.01×10^{-1}	2.24×10^{-2}	4.02×10^{-3}	2.63×10^{-8}	-2.65×10^{-2}	3.07×10^{-2}	3.88×10^{-1}
rs2652447	7.39×10^{-4}	4.90×10^{-3}	8.80×10^{-1}	2.01×10^{-2}	3.60×10^{-3}	2.46×10^{-8}	5.70×10^{-2}	2.76×10^{-2}	3.90×10^{-2}
rs2678908	-2.22×10^{-3}	4.91×10^{-3}	6.50×10^{-1}	1.99×10^{-2}	3.64×10^{-3}	4.48×10^{-8}	-3.61×10^{-2}	2.77×10^{-2}	1.93×10^{-1}
rs2867105	-2.91×10^{-2}	6.32×10^{-3}	4.10×10^{-6}	3.17×10^{-2}	4.65×10^{-3}	9.21×10^{-12}	9.80×10^{-2}	3.57×10^{-2}	6.09×10^{-3}
rs324769	2.68×10^{-2}	4.83×10^{-3}	3.05×10^{-8}	-1.44×10^{-2}	3.57×10^{-3}	5.09×10^{-5}	-1.20×10^{-3}	2.75×10^{-2}	9.65×10^{-1}
rs357512	1.04×10^{-2}	5.54×10^{-3}	6.03×10^{-2}	2.23×10^{-2}	4.07×10^{-3}	4.07×10^{-8}	-3.88×10^{-2}	3.11×10^{-2}	2.12×10^{-1}
rs3766823	-3.94×10^{-2}	6.39×10^{-3}	7.09×10^{-10}	2.14×10^{-2}	4.70×10^{-3}	5.22×10^{-6}	2.82×10^{-2}	3.63×10^{-2}	4.37×10^{-1}
rs3795310	-9.69×10^{-3}	4.83×10^{-3}	4.50×10^{-2}	-2.03×10^{-2}	3.58×10^{-3}	1.45×10^{-8}	-2.80×10^{-3}	2.73×10^{-2}	9.18×10^{-1}
rs3800227	-8.40×10^{-3}	5.52×10^{-3}	1.28×10^{-1}	2.28×10^{-2}	4.06×10^{-3}	1.93×10^{-8}	6.18×10^{-2}	3.09×10^{-2}	4.54×10^{-2}
rs3828917	-6.71×10^{-2}	1.20×10^{-2}	2.27×10^{-8}	3.82×10^{-3}	9.70×10^{-3}	6.93×10^{-1}	4.60×10^{-2}	7.70×10^{-2}	5.50×10^{-1}
rs3857914	-8.59×10^{-3}	5.26×10^{-3}	1.02×10^{-1}	2.54×10^{-2}	3.86×10^{-3}	4.46×10^{-11}	-1.54×10^{-2}	2.93×10^{-2}	5.99×10^{-1}
rs3897990	-1.73×10^{-2}	5.59×10^{-3}	1.98×10^{-3}	2.33×10^{-2}	4.19×10^{-3}	2.60×10^{-8}	4.42×10^{-2}	3.15×10^{-2}	1.61×10^{-1}
rs4634524	3.48×10^{-2}	5.87×10^{-3}	2.92×10^{-9}	-1.58×10^{-2}	4.36×10^{-3}	2.81×10^{-4}	6.90×10^{-3}	3.27×10^{-2}	8.33×10^{-1}
rs4713692	2.76×10^{-2}	4.99×10^{-3}	3.07×10^{-8}	1.56×10^{-3}	3.71×10^{-3}	6.73×10^{-1}	-2.94×10^{-2}	2.83×10^{-2}	3.00×10^{-1}

SNPs	GERD			Smoking Initiation			IPF		
	Beta	SE	*P*-value	Beta	SE	*P*-value	Beta	SE	*P*-value
rs4731315	3.20×10^{-2}	5.73×10^{-3}	2.39×10^{-8}	-9.29×10^{-3}	4.26×10^{-3}	2.93×10^{-2}	6.01×10^{-2}	3.28×10^{-2}	6.67×10^{-2}
rs4781977	3.84×10^{-3}	5.79×10^{-3}	5.07×10^{-1}	-2.39×10^{-2}	4.36×10^{-3}	4.54×10^{-8}	-2.69×10^{-2}	3.32×10^{-2}	4.18×10^{-1}
rs4869748	2.88×10^{-2}	5.00×10^{-3}	8.79×10^{-9}	-8.17×10^{-3}	3.70×10^{-3}	2.75×10^{-2}	1.70×10^{-3}	2.83×10^{-2}	9.52×10^{-1}
rs569356	3.79×10^{-2}	6.91×10^{-3}	4.07×10^{-8}	4.44×10^{-3}	5.20×10^{-3}	3.93×10^{-1}	-3.39×10^{-2}	3.90×10^{-2}	3.85×10^{-1}
rs6050446	-4.86×10^{-2}	1.35×10^{-2}	3.21×10^{-4}	5.83×10^{-2}	1.03×10^{-2}	1.56×10^{-8}	6.96×10^{-2}	8.05×10^{-2}	3.87×10^{-1}
rs6415872	3.58×10^{-3}	4.82×10^{-3}	4.58×10^{-1}	-2.37×10^{-2}	3.56×10^{-3}	2.49×10^{-11}	-1.81×10^{-2}	2.71×10^{-2}	5.04×10^{-1}
rs6476180	-7.33×10^{-3}	4.81×10^{-3}	1.28×10^{-1}	1.97×10^{-2}	3.56×10^{-3}	2.95×10^{-8}	-1.78×10^{-2}	2.72×10^{-2}	5.13×10^{-1}
rs6597540	2.83×10^{-2}	5.16×10^{-3}	4.04×10^{-8}	-1.85×10^{-2}	3.81×10^{-3}	1.21×10^{-6}	2.29×10^{-2}	2.90×10^{-2}	4.29×10^{-1}
rs6656687	-7.82×10^{-3}	4.83×10^{-3}	1.05×10^{-1}	1.99×10^{-2}	3.56×10^{-3}	2.36×10^{-8}	-3.00×10^{-2}	2.72×10^{-2}	2.70×10^{-1}
rs6717424	-5.82×10^{-3}	4.85×10^{-3}	2.30×10^{-1}	2.74×10^{-2}	3.60×10^{-3}	2.63×10^{-14}	3.62×10^{-2}	2.76×10^{-2}	1.90×10^{-1}
rs6748263	-2.63×10^{-2}	4.83×10^{-3}	4.95×10^{-8}	1.36×10^{-2}	3.56×10^{-3}	1.40×10^{-4}	-4.88×10^{-2}	2.96×10^{-2}	9.94×10^{-2}
rs6750529	-1.35×10^{-2}	5.46×10^{-3}	1.36×10^{-2}	2.24×10^{-2}	4.06×10^{-3}	3.54×10^{-8}	-1.05×10^{-2}	3.12×10^{-2}	7.36×10^{-1}
rs715693	-1.41×10^{-2}	4.97×10^{-3}	4.68×10^{-3}	2.00×10^{-2}	3.65×10^{-3}	4.44×10^{-8}	-1.47×10^{-2}	2.79×10^{-2}	5.99×10^{-1}
rs7238242	1.12×10^{-3}	4.86×10^{-3}	8.18×10^{-1}	-2.04×10^{-2}	3.59×10^{-3}	1.27×10^{-8}	-3.75×10^{-2}	2.73×10^{-2}	1.69×10^{-1}
rs7258722	2.77×10^{-2}	4.92×10^{-3}	1.81×10^{-8}	8.44×10^{-4}	3.63×10^{-3}	8.16×10^{-1}	5.50×10^{-3}	2.77×10^{-2}	8.43×10^{-1}
rs7283473	-2.94×10^{-2}	5.18×10^{-3}	1.44×10^{-8}	-5.44×10^{-3}	3.86×10^{-3}	1.59×10^{-1}	4.00×10^{-3}	2.95×10^{-2}	8.92×10^{-1}
rs7303854	-3.02×10^{-2}	5.15×10^{-3}	4.41×10^{-9}	3.22×10^{-3}	3.78×10^{-3}	3.94×10^{-1}	-5.25×10^{-2}	2.89×10^{-2}	6.91×10^{-2}
rs7319102	3.89×10^{-2}	5.75×10^{-3}	1.33×10^{-11}	-7.78×10^{-3}	4.24×10^{-3}	6.65×10^{-2}	6.80×10^{-3}	3.55×10^{-2}	8.48×10^{-1}
rs7357604	-2.88×10^{-2}	4.92×10^{-3}	4.57×10^{-9}	-6.52×10^{-3}	3.63×10^{-3}	7.28×10^{-2}	3.90×10^{-3}	2.78×10^{-2}	8.89×10^{-1}
rs7556896	-3.33×10^{-2}	5.18×10^{-3}	1.27×10^{-10}	6.28×10^{-3}	3.84×10^{-3}	1.02×10^{-1}	2.39×10^{-2}	2.95×10^{-2}	4.18×10^{-1}
rs7612999	-3.05×10^{-2}	5.60×10^{-3}	4.90×10^{-8}	4.27×10^{-3}	4.11×10^{-3}	2.99×10^{-1}	-2.27×10^{-2}	3.13×10^{-2}	4.68×10^{-1}
rs761777	-3.45×10^{-2}	5.54×10^{-3}	4.71×10^{-10}	1.05×10^{-2}	4.12×10^{-3}	1.11×10^{-2}	8.30×10^{-3}	3.09×10^{-2}	7.88×10^{-1}

**SNPs**		**GERD**			**Smoking Initiation**			**IPF**	
	**Beta**	**SE**	***P*-value**	**Beta**	**SE**	***P*-value**	**Beta**	**SE**	***P*-value**
rs7628120	3.04×10^{-2}	5.37×10^{-3}	1.53×10^{-8}	3.00×10^{-3}	3.93×10^{-3}	4.45×10^{-1}	4.62×10^{-2}	2.97×10^{-2}	1.20×10^{-1}
rs7659565	-3.27×10^{-2}	5.96×10^{-3}	4.30×10^{-8}	4.92×10^{-3}	4.40×10^{-3}	2.64×10^{-1}	-2.50×10^{-2}	3.32×10^{-2}	4.51×10^{-1}
rs7701346	2.72×10^{-2}	4.89×10^{-3}	2.74×10^{-8}	-5.80×10^{-3}	3.60×10^{-3}	1.07×10^{-1}	-1.00×10^{-4}	2.74×10^{-2}	9.97×10^{-1}
rs772030	1.95×10^{-2}	4.93×10^{-3}	7.81×10^{-5}	-2.15×10^{-2}	3.62×10^{-3}	3.03×10^{-9}	-1.80×10^{-2}	2.78×10^{-2}	5.18×10^{-1}
rs7844990	4.44×10^{-3}	4.92×10^{-3}	3.67×10^{-1}	2.01×10^{-2}	3.63×10^{-3}	3.23×10^{-8}	3.85×10^{-2}	2.76×10^{-2}	1.63×10^{-1}
rs7901883	6.96×10^{-3}	5.73×10^{-3}	2.25×10^{-1}	-2.45×10^{-2}	4.25×10^{-3}	7.98×10^{-9}	2.16×10^{-2}	3.21×10^{-2}	5.02×10^{-1}
rs7942368	3.40×10^{-2}	5.92×10^{-3}	9.54×10^{-9}	-7.26×10^{-3}	4.31×10^{-3}	9.17×10^{-2}	-1.97×10^{-2}	3.28×10^{-2}	5.48×10^{-1}
rs808719	-2.78×10^{-2}	4.82×10^{-3}	8.48×10^{-9}	1.52×10^{-2}	3.56×10^{-3}	1.92×10^{-5}	-1.48×10^{-2}	2.71×10^{-2}	5.85×10^{-1}
rs861575	-2.76×10^{-2}	4.89×10^{-3}	1.63×10^{-8}	4.70×10^{-3}	3.60×10^{-3}	1.91×10^{-1}	2.91×10^{-2}	2.75×10^{-2}	2.90×10^{-1}
rs903678	-2.77×10^{-2}	5.08×10^{-3}	4.89×10^{-8}	7.06×10^{-3}	3.76×10^{-3}	6.05×10^{-2}	-9.70×10^{-2}	2.89×10^{-2}	7.37×10^{-1}
rs917154	2.56×10^{-2}	6.66×10^{-3}	1.24×10^{-4}	-2.91×10^{-2}	4.90×10^{-3}	2.77×10^{-9}	3.35×10^{-2}	3.72×10^{-2}	3.68×10^{-1}
rs9322173	1.90×10^{-2}	4.81×10^{-3}	8.26×10^{-5}	-1.96×10^{-2}	3.56×10^{-3}	3.74×10^{-8}	2.66×10^{-2}	2.94×10^{-2}	3.66×10^{-1}
rs9373363	3.27×10^{-2}	5.56×10^{-3}	4.13×10^{-9}	-1.40×10^{-2}	4.07×10^{-3}	5.95×10^{-4}	4.43×10^{-2}	3.16×10^{-2}	1.61×10^{-1}
rs948536	-3.29×10^{-2}	5.76×10^{-3}	1.12×10^{-8}	5.32×10^{-3}	4.24×10^{-3}	2.10×10^{-1}	8.12×10^{-2}	3.20×10^{-2}	1.13×10^{-1}
rs9529119	-3.22×10^{-2}	5.79×10^{-3}	2.61×10^{-8}	4.59×10^{-3}	4.26×10^{-3}	2.81×10^{-1}	5.26×10^{-2}	3.28×10^{-2}	1.09×10^{-1}
rs9540718	-2.67×10^{-2}	4.82×10^{-3}	3.04×10^{-8}	1.86×10^{-2}	3.56×10^{-3}	1.64×10^{-7}	1.49×10^{-2}	2.72×10^{-2}	5.84×10^{-1}
rs9615905	-2.76×10^{-2}	4.84×10^{-3}	1.21×10^{-8}	6.04×10^{-3}	3.58×10^{-3}	9.13×10^{-2}	7.60×10^{-3}	2.72×10^{-2}	7.80×10^{-1}
rs9930501	-2.93×10^{-2}	4.85×10^{-3}	1.41×10^{-9}	4.75×10^{-3}	3.58×10^{-3}	1.84×10^{-1}	-2.46×10^{-2}	2.73×10^{-2}	3.67×10^{-1}
**Replication Cohort (number of SNPs: 195)**									
rs10036561	1.38×10^{-2}	1.00×10^{-2}	1.67×10^{-1}	2.35 × 10-2	4.08×10^{-3}	9.36×10^{-9}	-2.40×10^{-2}	3.45×10^{-2}	4.87×10^{-1}
rs10075489	1.41×10^{-2}	9.82×10^{-3}	1.51×10^{-1}	1.88 × 10-2	3.80×10^{-3}	8.19×10^{-7}	1.74×10^{-2}	3.39×10^{-2}	6.09×10^{-1}
rs1008078	-7.42×10^{-3}	9.29×10^{-2}	4.25×10^{-1}	2.22 × 10-2	3.63×10^{-3}	8.61×10^{-10}	1.35×10^{-2}	3.21×10^{-2}	6.74×10^{-1}

SNPs	GERD			Smoking Initiation			IPF		
	**Beta**	**SE**	***P*-value**	**Beta**	**SE**	***P*-value**	**Beta**	**SE**	***P*-value**
rs10105293	-1.41×10^{-2}	9.23×10^{-3}	1.27×10^{-1}	-2.47×10^{-2}	3.64×10^{-3}	1.09×10^{-11}	-6.42×10^{-2}	3.19×10^{-2}	4.42×10^{-2}
rs10201987	5.81×10^{-3}	9.19×10^{-3}	5.28×10^{-1}	1.83×10^{-2}	3.75×10^{-3}	9.72×10^{-7}	-2.90×10^{-2}	3.19×10^{-2}	3.63×10^{-1}
rs10249981	-2.88×10^{-2}	1.00×10^{-2}	4.13×10^{-3}	-1.81×10^{-2}	3.70×10^{-3}	9.68×10^{-7}	-3.20×10^{-3}	3.50×10^{-2}	9.27×10^{-1}
rs10490159	1.13×10^{-2}	9.52×10^{-3}	2.35×10^{-1}	2.16×10^{-2}	3.63×10^{-3}	2.59×10^{-9}	-1.38×10^{-2}	3.30×10^{-2}	6.76×10^{-1}
rs10498846	1.13×10^{-2}	9.21×10^{-3}	2.18×10^{-1}	2.06×10^{-2}	3.56×10^{-3}	6.62×10^{-9}	1.04×10^{-2}	3.19×10^{-2}	7.45×10^{-1}
rs10830924	-2.44×10^{-3}	9.29×10^{-3}	7.93×10^{-1}	1.81×10^{-2}	3.58×10^{-3}	4.17×10^{-7}	4.55×10^{-2}	3.22×10^{-2}	1.57×10^{-1}
rs10907322	-7.55×10^{-3}	9.21×10^{-3}	4.13×10^{-1}	-1.76×10^{-2}	3.56×10^{-3}	7.52×10^{-7}	5.94×10^{-3}	3.19×10^{-2}	8.52×10^{-1}
rs10953957	2.04×10^{-2}	9.38×10^{-3}	2.94×10^{-2}	1.85×10^{-2}	3.66×10^{-3}	4.72×10^{-7}	3.51×10^{-3}	3.25×10^{-2}	9.14×10^{-1}
rs10959411	-1.99×10^{-2}	1.13×10^{-2}	7.82×10^{-2}	-2.43×10^{-2}	4.90×10^{-3}	7.41×10^{-7}	1.16×10^{-2}	3.88×10^{-2}	7.65×10^{-1}
rs11030380	-8.95×10^{-3}	9.43×10^{-3}	3.42×10^{-1}	2.01×10^{-2}	3.63×10^{-3}	2.97×10^{-8}	-1.51×10^{-3}	3.26×10^{-2}	9.63×10^{-1}
rs11073474	-3.32×10^{-2}	9.83×10^{-3}	7.22×10^{-4}	-1.98×10^{-2}	4.01×10^{-3}	7.45×10^{-7}	2.87×10^{-2}	3.39×10^{-2}	3.97×10^{-1}
rs11076967	-1.87×10^{-2}	1.05×10^{-2}	7.58×10^{-2}	2.05×10^{-2}	4.14×10^{-3}	7.34×10^{-7}	-3.25×10^{-2}	3.67×10^{-2}	3.76×10^{-1}
rs1109480	-9.37×10^{-4}	9.69×10^{-3}	9.23×10^{-1}	-1.88×10^{-2}	3.65×10^{-3}	2.64×10^{-7}	-2.21×10^{-3}	3.34×10^{-2}	9.47×10^{-1}
rs1126757	-1.57×10^{-2}	9.26×10^{-3}	8.95×10^{-2}	1.87×10^{-2}	3.56×10^{-3}	1.53×10^{-7}	2.53×10^{-3}	3.21×10^{-2}	9.37×10^{-1}
rs113230003	-1.77×10^{-2}	1.12×10^{-2}	1.14×10^{-1}	-2.06×10^{-2}	4.09×10^{-3}	5.19×10^{-7}	1.26×10^{-1}	3.78×10^{-2}	8.74×10^{-4}
rs11675462	1.18×10^{-2}	1.08×10^{-2}	2.75×10^{-1}	2.01×10^{-2}	4.07×10^{-3}	7.69×10^{-7}	3.60×10^{-3}	3.75×10^{-2}	9.23×10^{-1}
rs11692435	-1.82×10^{-2}	1.10×10^{-2}	9.81×10^{-2}	3.04×10^{-2}	6.18×10^{-3}	8.71×10^{-7}	1.54×10^{-3}	3.76×10^{-2}	9.67×10^{-1}
rs117143374	2.09×10^{-2}	1.50×10^{-2}	1.63×10^{-1}	2.93×10^{-2}	5.27×10^{-3}	2.76×10^{-8}	-9.06×10^{-3}	5.25×10^{-2}	8.63×10^{-1}
rs11716779	-4.94×10^{-3}	1.18×10^{-2}	6.76×10^{-1}	2.20×10^{-2}	4.03×10^{-3}	4.88×10^{-8}	4.24×10^{-2}	4.13×10^{-2}	3.05×10^{-1}
rs117582306	5.49×10^{-3}	4.17×10^{-2}	8.95×10^{-1}	6.44×10^{-2}	1.23×10^{-2}	1.72×10^{-7}	3.17×10^{-1}	1.48×10^{-1}	3.20×10^{-2}
rs11771695	9.62×10^{-4}	9.65×10^{-3}	9.21×10^{-1}	-1.86×10^{-2}	3.74×10^{-3}	7.23×10^{-7}	2.87×10^{-2}	3.34×10^{-2}	3.91×10^{-1}
rs12036473	2.47×10^{-3}	9.29×10^{-3}	7.90×10^{-1}	1.90×10^{-2}	3.66×10^{-3}	2.31×10^{-7}	4.92×10^{-2}	3.21×10^{-2}	1.26×10^{-1}

**SNPs**	**GERD**			**Smoking Initiation**			**IPF**		
	**Beta**	**SE**	***P*-value**	**Beta**	**SE**	***P*-value**	**Beta**	**SE**	***P*-value**
rs12047884	1.05×10^{-2}	9.22×10^{-3}	2.53×10^{-1}	-1.76×10^{-2}	3.56×10^{-3}	7.53×10^{-7}	7.43×10^{-3}	3.19×10^{-2}	8.16×10^{-1}
rs12143162	2.49×10^{-3}	1.16×10^{-2}	8.30×10^{-1}	2.27×10^{-2}	4.50×10^{-3}	4.27×10^{-7}	2.75×10^{-2}	3.99×10^{-2}	4.90×10^{-1}
rs12208141	-1.73×10^{-2}	9.35×10^{-3}	6.45×10^{-2}	-1.79×10^{-2}	3.63×10^{-3}	7.79×10^{-7}	3.27×10^{-2}	3.23×10^{-2}	3.12×10^{-1}
rs12212878	-4.03×10^{-2}	2.95×10^{-2}	1.71×10^{-1}	-3.54×10^{-2}	7.20×10^{-3}	8.61×10^{-7}	1.68×10^{-1}	1.03×10^{-1}	1.02×10^{-1}
rs12238219	5.02×10^{-2}	9.93×10^{-3}	4.25×10^{-7}	-3.95×10^{-3}	4.13×10^{-3}	3.39×10^{-1}	-5.27×10^{-2}	3.45×10^{-2}	1.26×10^{-1}
rs12346687	-1.81×10^{-3}	9.40×10^{-3}	8.47×10^{-1}	1.83×10^{-2}	3.70×10^{-3}	7.43×10^{-7}	-1.43×10^{-2}	3.26×10^{-2}	6.60×10^{-1}
rs12442894	8.94×10^{-3}	1.16×10^{-2}	4.39×10^{-1}	-2.44×10^{-2}	4.29×10^{-3}	1.28×10^{-8}	-4.70×10^{-2}	4.01×10^{-2}	2.41×10^{-1}
rs12450454	7.76×10^{-3}	9.26×10^{-3}	4.02×10^{-1}	-2.00×10^{-2}	3.61×10^{-3}	3.04×10^{-8}	-5.62×10^{-2}	3.21×10^{-2}	7.97×10^{-2}
rs12450454	1.18×10^{-1}	1.33×10^{-1}	3.76×10^{-1}	-2.00×10^{-2}	3.61×10^{-3}	3.04×10^{-8}	1.28×10^{-2}	4.38×10^{-1}	9.77×10^{-1}
rs1248860	-1.47×10^{-2}	9.27×10^{-3}	1.12×10^{-1}	1.75×10^{-2}	3.57×10^{-3}	9.73×10^{-7}	-1.58×10^{-2}	3.21×10^{-2}	6.24×10^{-1}
rs12539177	2.34×10^{-3}	9.43×10^{-3}	8.04×10^{-1}	1.84×10^{-2}	3.70×10^{-3}	6.09×10^{-7}	4.16×10^{-2}	3.27×10^{-2}	2.03×10^{-1}
rs12612538	4.90×10^{-2}	9.45×10^{-3}	2.23×10^{-7}	-4.96×10^{-3}	3.65×10^{-3}	1.74×10^{-1}	2.23×10^{-4}	3.29×10^{-2}	9.95×10^{-1}
rs12770588	2.17×10^{-2}	1.23×10^{-2}	7.71×10^{-2}	2.33×10^{-2}	4.61×10^{-3}	4.44×10^{-7}	-6.93×10^{-4}	4.25×10^{-2}	9.87×10^{-1}
rs12783374	1.04×10^{-2}	1.04×10^{-2}	3.16×10^{-1}	2.06×10^{-2}	4.04×10^{-3}	3.65×10^{-7}	4.37×10^{-3}	3.59×10^{-2}	9.03×10^{-1}
rs12786130	7.56×10^{-3}	1.11×10^{-2}	4.94×10^{-1}	-2.22×10^{-2}	4.15×10^{-3}	9.46×10^{-8}	-1.31×10^{-2}	3.85×10^{-2}	7.33×10^{-1}
rs12874552	1.45×10^{-2}	9.71×10^{-3}	1.36×10^{-1}	-1.97×10^{-2}	3.60×10^{-3}	4.24×10^{-8}	4.15×10^{-2}	3.37×10^{-2}	2.18×10^{-1}
rs12880545	-6.48×10^{-3}	9.84×10^{-3}	5.10×10^{-1}	1.85×10^{-2}	3.70×10^{-3}	6.19×10^{-7}	-2.51×10^{-2}	3.40×10^{-2}	4.61×10^{-1}
rs12894851	-2.28×10^{-2}	1.44×10^{-2}	1.14×10^{-1}	-2.37×10^{-2}	4.78×10^{-3}	7.29×10^{-7}	-4.57×10^{-2}	5.02×10^{-2}	3.62×10^{-1}
rs13025009	-7.04×10^{-3}	1.52×10^{-2}	6.43×10^{-1}	2.18×10^{-2}	4.45×10^{-3}	9.61×10^{-7}	1.00×10^{-1}	5.39×10^{-2}	6.33×10^{-2}
rs13025213	1.24×10^{-2}	1.08×10^{-2}	2.51×10^{-1}	2.04×10^{-2}	4.02×10^{-3}	3.98×10^{-7}	3.12×10^{-2}	3.74×10^{-2}	4.04×10^{-1}
rs13136052	-9.14×10^{-3}	9.38×10^{-3}	3.30×10^{-1}	-1.98×10^{-2}	3.59×10^{-3}	3.35×10^{-8}	-2.27×10^{-2}	3.26×10^{-2}	4.86×10^{-1}
rs13140558	5.95×10^{-2}	1.20×10^{-2}	7.84×10^{-7}	3.29×10^{-3}	4.22×10^{-3}	4.36×10^{-1}	-5.63×10^{-3}	4.22×10^{-2}	8.94×10^{-1}

SNPs		GERD			Smoking Initiation			IPF	
	Beta	SE	***P*-value**	Beta	SE	***P*-value**	Beta	SE	***P*-value**
rs13391128	1.96×10^{-2}	1.44×10^{-2}	1.75×10^{-1}	2.79×10^{-2}	5.34×10^{-3}	1.70×10^{-7}	-4.85×10^{-2}	5.02×10^{-2}	3.34×10^{-1}
rs1343424	-1.87×10^{-3}	1.15×10^{-2}	8.71×10^{-1}	2.28×10^{-2}	4.17×10^{-3}	4.67×10^{-8}	1.76×10^{-2}	4.00×10^{-2}	6.60×10^{-1}
rs134529	-5.28×10^{-3}	9.79×10^{-3}	5.90×10^{-1}	-2.00×10^{-2}	3.66×10^{-3}	4.85×10^{-8}	1.91×10^{-2}	3.38×10^{-2}	5.72×10^{-1}
rs1350035	-2.61×10^{-2}	9.23×10^{-3}	4.70×10^{-3}	-1.80×10^{-2}	3.65×10^{-3}	8.16×10^{-7}	-1.03×10^{-1}	3.18×10^{-2}	1.14×10^{-3}
rs1392510	-1.04×10^{-2}	1.25×10^{-2}	4.05×10^{-1}	2.76×10^{-2}	5.11×10^{-3}	6.84×10^{-8}	-1.96×10^{-2}	4.27×10^{-2}	6.46×10^{-1}
rs1427499	-1.56×10^{-3}	9.95×10^{-3}	8.75×10^{-1}	-2.24×10^{-2}	3.92×10^{-3}	1.08×10^{-8}	-2.58×10^{-2}	3.45×10^{-2}	4.54×10^{-1}
rs1466802	3.09×10^{-2}	1.11×10^{-2}	5.26×10^{-3}	2.35×10^{-2}	4.21×10^{-3}	2.21×10^{-8}	3.14×10^{-2}	3.82×10^{-2}	4.10×10^{-1}
rs147662818	4.04×10^{-2}	2.19×10^{-2}	6.58×10^{-2}	1.02×10^{-1}	1.90×10^{-2}	7.25×10^{-8}	-1.81×10^{-2}	7.47×10^{-2}	8.08×10^{-1}
rs1505800	-1.65×10^{-3}	1.10×10^{-2}	8.81×10^{-1}	-2.01×10^{-2}	4.02×10^{-3}	6.15×10^{-7}	2.86×10^{-2}	3.85×10^{-2}	4.57×10^{-1}
rs152567	2.03×10^{-2}	9.95×10^{-3}	4.14×10^{-2}	-2.01×10^{-2}	3.70×10^{-3}	5.93×10^{-8}	-3.94×10^{-3}	3.46×10^{-2}	9.09×10^{-1}
rs1545506	1.52×10^{-2}	9.24×10^{-3}	1.00×10^{-1}	1.80×10^{-2}	3.56×10^{-3}	4.41×10^{-7}	5.52×10^{-2}	3.19×10^{-2}	8.36×10^{-2}
rs17144411	1.59×10^{-2}	1.33×10^{-2}	2.34×10^{-1}	2.32×10^{-2}	4.64×10^{-3}	5.52×10^{-7}	3.53×10^{-2}	4.63×10^{-2}	4.46×10^{-1}
rs17403088	7.36×10^{-3}	1.25×10^{-2}	5.55×10^{-1}	-2.27×10^{-2}	4.64×10^{-3}	9.48×10^{-7}	5.17×10^{-2}	4.34×10^{-2}	2.34×10^{-1}
rs17613129	2.78×10^{-2}	1.01×10^{-2}	6.20×10^{-3}	2.19×10^{-2}	3.90×10^{-3}	1.89×10^{-8}	8.88×10^{-3}	3.53×10^{-2}	8.01×10^{-1}
rs1805871	1.49×10^{-2}	9.35×10^{-3}	1.12×10^{-1}	1.85×10^{-2}	3.76×10^{-3}	8.10×10^{-7}	3.80×10^{-2}	3.23×10^{-2}	2.39×10^{-1}
rs1841252	-2.74×10^{-2}	9.43×10^{-3}	3.68×10^{-3}	-1.88×10^{-2}	3.56×10^{-3}	1.23×10^{-7}	-4.90×10^{-2}	3.26×10^{-2}	1.33×10^{-1}
rs1853599	-8.97×10^{-3}	9.76×10^{-3}	3.58×10^{-1}	2.03×10^{-2}	3.77×10^{-3}	7.39×10^{-8}	5.74×10^{-2}	3.38×10^{-2}	8.91×10^{-2}
rs1856047	2.00×10^{-3}	9.45×10^{-3}	8.33×10^{-1}	-1.87×10^{-2}	3.58×10^{-3}	1.97×10^{-7}	5.33×10^{-2}	3.28×10^{-2}	1.04×10^{-1}
rs1895947	3.87×10^{-3}	1.06×10^{-2}	7.15×10^{-1}	2.06×10^{-2}	4.17×10^{-3}	8.46×10^{-7}	5.88×10^{-3}	3.65×10^{-2}	8.72×10^{-1}
rs1898428	9.72×10^{-4}	9.25×10^{-3}	9.16×10^{-1}	1.78×10^{-2}	3.56×10^{-3}	5.31×10^{-7}	4.14×10^{-2}	3.21×10^{-2}	1.97×10^{-1}
rs1899492	1.09×10^{-2}	9.23×10^{-3}	2.38×10^{-1}	1.86×10^{-2}	3.56×10^{-3}	1.87×10^{-7}	-8.19×10^{-2}	3.19×10^{-2}	1.04×10^{-2}
rs1910236	1.24×10^{-3}	9.23×10^{-3}	8.93×10^{-1}	1.92×10^{-2}	3.56×10^{-3}	6.44×10^{-8}	-3.88×10^{-2}	3.19×10^{-2}	2.24×10^{-1}

SNPs	GERD			Smoking Initiation			IPF		
	**Beta**	**SE**	***P*-value**	**Beta**	**SE**	***P*-value**	**Beta**	**SE**	***P*-value**
rs1935958	-1.42×10^{-2}	1.40×10^{-2}	3.12×10^{-1}	-2.47×10^{-2}	4.95×10^{-3}	6.45×10^{-7}	-3.49×10^{-2}	4.89×10^{-2}	4.76×10^{-1}
rs2017500	1.02×10^{-3}	9.23×10^{-3}	9.12×10^{-1}	1.79×10^{-2}	3.56×10^{-3}	4.96×10^{-7}	-2.29×10^{-2}	3.20×10^{-2}	4.74×10^{-1}
rs2035284	1.10×10^{-2}	1.04×10^{-2}	2.90×10^{-1}	-1.89×10^{-2}	3.86×10^{-3}	9.77×10^{-7}	3.50×10^{-2}	3.61×10^{-2}	3.31×10^{-1}
rs2058799	3.15×10^{-3}	9.21×10^{-3}	7.32×10^{-1}	1.92×10^{-2}	3.60×10^{-3}	9.32×10^{-8}	-6.65×10^{-2}	3.19×10^{-2}	8.35×10^{-1}
rs2074640	-7.90×10^{-3}	1.11×10^{-2}	4.76×10^{-1}	1.94×10^{-2}	3.89×10^{-3}	5.88×10^{-7}	-6.34×10^{-3}	3.85×10^{-2}	8.69×10^{-1}
rs2120459	8.11×10^{-3}	1.09×10^{-2}	4.55×10^{-1}	1.94×10^{-2}	3.91×10^{-3}	7.36×10^{-7}	-4.08×10^{-2}	3.77×10^{-2}	2.80×10^{-1}
rs2173019	3.58×10^{-3}	1.35×10^{-2}	7.90×10^{-1}	2.40×10^{-2}	4.68×10^{-3}	2.76×10^{-7}	2.39×10^{-2}	4.68×10^{-2}	6.09×10^{-1}
rs2256027	6.29×10^{-2}	3.50×10^{-2}	7.21×10^{-2}	5.42×10^{-2}	1.06×10^{-3}	3.61×10^{-7}	1.11×10^{-1}	1.19×10^{-1}	3.50×10^{-1}
rs2281756	-4.59×10^{-3}	9.32×10^{-3}	6.22×10^{-1}	-2.01×10^{-2}	3.61×10^{-3}	2.71×10^{-7}	-1.08×10^{-2}	3.22×10^{-2}	7.37×10^{-1}
rs2435207	6.98×10^{-3}	9.37×10^{-3}	4.56×10^{-1}	1.88×10^{-2}	3.80×10^{-3}	7.36×10^{-7}	4.12×10^{-2}	3.24×10^{-2}	2.04×10^{-1}
rs2551456	1.97×10^{-3}	9.32×10^{-3}	8.32×10^{-1}	1.79×10^{-2}	3.58×10^{-3}	5.50×10^{-7}	-4.27×10^{-2}	3.23×10^{-2}	1.86×10^{-1}
rs2591152	2.65×10^{-3}	9.29×10^{-3}	7.76×10^{-1}	1.89×10^{-2}	3.59×10^{-3}	1.50×10^{-7}	6.29×10^{-2}	3.21×10^{-2}	5.05×10^{-2}
rs2675609	9.29×10^{-3}	9.46×10^{-3}	3.26×10^{-1}	-1.91×10^{-2}	3.67×10^{-3}	1.95×10^{-7}	3.06×10^{-2}	3.27×10^{-2}	3.49×10^{-1}
rs2710634	-2.31×10^{-2}	9.24×10^{-3}	1.22×10^{-2}	-1.82×10^{-2}	3.56×10^{-3}	3.07×10^{-7}	2.09×10^{-2}	3.20×10^{-2}	5.13×10^{-1}
rs2717068	1.25×10^{-2}	9.50×10^{-3}	1.89×10^{-1}	1.81×10^{-2}	3.65×10^{-3}	7.57×10^{-7}	8.29×10^{-3}	3.29×10^{-2}	8.01×10^{-1}
rs2730850	1.40×10^{-3}	1.33×10^{-2}	9.16×10^{-1}	2.63×10^{-2}	5.38×10^{-3}	9.95×10^{-7}	8.42×10^{-4}	4.59×10^{-2}	9.85×10^{-1}
rs284843	1.02×10^{-3}	9.61×10^{-3}	9.16×10^{-1}	-2.00×10^{-2}	4.08×10^{-3}	9.42×10^{-7}	-2.57×10^{-2}	3.32×10^{-2}	4.38×10^{-1}
rs28585984	-1.03×10^{-2}	1.02×10^{-2}	3.13×10^{-1}	-1.92×10^{-2}	3.86×10^{-3}	6.66×10^{-7}	-5.57×10^{-2}	3.53×10^{-2}	1.15×10^{-1}
rs2867105	-1.28×10^{-2}	1.22×10^{-2}	2.95×10^{-1}	3.17×10^{-2}	4.65×10^{-3}	9.21×10^{-12}	2.87×10^{-2}	4.26×10^{-2}	5.01×10^{-1}
rs293055	1.28×10^{-2}	9.26×10^{-3}	1.67×10^{-1}	2.17×10^{-2}	3.56×10^{-3}	1.17×10^{-9}	3.84×10^{-2}	3.21×10^{-2}	2.31×10^{-1}
rs2952251	1.29×10^{-2}	1.10×10^{-2}	2.38×10^{-1}	2.21×10^{-2}	4.29×10^{-3}	2.51×10^{-7}	4.32×10^{-2}	3.81×10^{-2}	2.56×10^{-1}
rs2958853	-4.13×10^{-3}	9.20×10^{-3}	6.53×10^{-1}	-1.77×10^{-2}	3.56×10^{-3}	6.40×10^{-7}	3.71×10^{-2}	3.18×10^{-2}	2.44×10^{-1}

SNPs	GERD			Smoking Initiation			IPF		
	**Beta**	**SE**	***P*-value**	**Beta**	**SE**	***P*-value**	**Beta**	**SE**	***P*-value**
rs2959100	8.52×10^{-3}	9.22×10^{-3}	3.55×10^{-1}	-1.87×10^{-2}	3.58×10^{-3}	1.75×10^{-7}	2.44×10^{-2}	3.19×10^{-2}	4.45×10^{-1}
rs310333	4.87×10^{-4}	9.89×10^{-3}	9.61×10^{-1}	1.96×10^{-2}	3.95×10^{-3}	6.65×10^{-7}	5.46×10^{-2}	3.42×10^{-2}	1.11×10^{-1}
rs317630	-3.50×10^{-3}	9.61×10^{-3}	7.16×10^{-1}	-2.25×10^{-2}	3.97×10^{-3}	1.49×10^{-8}	6.15×10^{-2}	3.33×10^{-2}	6.48×10^{-2}
rs3213876	-2.51×10^{-2}	1.00×10^{-2}	1.21×10^{-2}	1.93×10^{-2}	3.73×10^{-3}	2.24×10^{-7}	-3.82×10^{-2}	3.48×10^{-2}	2.73×10^{-1}
rs324768	-2.67×10^{-2}	9.27×10^{-3}	4.05×10^{-3}	-1.81×10^{-2}	3.65×10^{-3}	6.92×10^{-7}	3.25×10^{-2}	3.21×10^{-2}	3.12×10^{-1}
rs34724099	7.20×10^{-3}	1.02×10^{-2}	4.82×10^{-1}	2.05×10^{-2}	3.95×10^{-3}	2.01×10^{-7}	8.38×10^{-3}	3.55×10^{-2}	8.13×10^{-1}
rs35733856	2.29×10^{-2}	9.22×10^{-3}	1.31×10^{-2}	1.91×10^{-2}	3.63×10^{-3}	1.32×10^{-7}	4.87×10^{-2}	3.19×10^{-2}	1.27×10^{-1}
rs35887108	-1.75×10^{-2}	1.02×10^{-2}	8.60×10^{-2}	-2.09×10^{-2}	4.27×10^{-3}	9.74×10^{-7}	-3.22×10^{-2}	3.52×10^{-2}	3.60×10^{-1}
rs36110110	3.84×10^{-1}	7.57×10^{-2}	3.86×10^{-7}	1.09×10^{-2}	1.81×10^{-2}	5.47×10^{-1}	-3.95×10^{-1}	3.09×10^{-1}	2.01×10^{-1}
rs3815195	6.07×10^{-3}	9.30×10^{-3}	5.14×10^{-1}	-1.77×10^{-2}	3.56×10^{-3}	6.81×10^{-7}	2.51×10^{-2}	3.22×10^{-2}	4.35×10^{-1}
rs41264285	3.18×10^{-3}	1.21×10^{-2}	7.92×10^{-1}	2.31×10^{-2}	4.28×10^{-3}	6.76×10^{-8}	-9.91×10^{-2}	4.19×10^{-2}	1.81×10^{-2}
rs41274959	-3.04×10^{-2}	5.27×10^{-2}	5.65×10^{-1}	-4.80×10^{-2}	9.22×10^{-3}	1.93×10^{-7}	6.62×10^{-2}	1.89×10^{-1}	7.26×10^{-1}
rs4269485	-4.88×10^{-2}	9.20×10^{-3}	1.10×10^{-7}	1.64×10^{-3}	3.83×10^{-3}	6.69×10^{-1}	-3.79×10^{-2}	3.20×10^{-2}	2.36×10^{-1}
rs4290831	1.53×10^{-2}	1.29×10^{-2}	2.38×10^{-1}	3.19×10^{-2}	6.31×10^{-3}	4.14×10^{-7}	-1.95×10^{-2}	4.42×10^{-2}	6.59×10^{-1}
rs430943	2.14×10^{-3}	1.06×10^{-2}	8.40×10^{-1}	2.46×10^{-2}	4.11×10^{-3}	2.13×10^{-9}	7.53×10^{-2}	3.67×10^{-2}	3.98×10^{-2}
rs4310854	-4.30×10^{-3}	9.27×10^{-3}	6.43×10^{-1}	-1.81×10^{-2}	3.63×10^{-3}	6.23×10^{-7}	3.48×10^{-2}	3.20×10^{-2}	2.77×10^{-1}
rs4471123	9.57×10^{-3}	9.18×10^{-3}	2.97×10^{-1}	1.99×10^{-2}	3.56×10^{-3}	2.23×10^{-8}	-2.80×10^{-2}	3.19×10^{-2}	3.80×10^{-1}
rs4480845	1.09×10^{-2}	9.54×10^{-3}	2.55×10^{-1}	1.82×10^{-2}	3.69×10^{-3}	8.65×10^{-7}	3.81×10^{-2}	3.31×10^{-2}	2.49×10^{-1}
rs4543050	-6.49×10^{-3}	1.33×10^{-2}	6.25×10^{-1}	2.60×10^{-2}	4.58×10^{-3}	1.39×10^{-8}	4.04×10^{-3}	4.61×10^{-2}	9.30×10^{-1}
rs4667082	5.83×10^{-3}	1.05×10^{-2}	5.78×10^{-1}	2.11×10^{-2}	4.08×10^{-3}	2.55×10^{-7}	-2.63×10^{-2}	3.63×10^{-2}	4.68×10^{-1}
rs4714070	9.35×10^{-3}	9.21×10^{-3}	3.10×10^{-1}	-1.76×10^{-2}	3.56×10^{-3}	7.58×10^{-7}	3.53×10^{-2}	3.19×10^{-2}	2.69×10^{-1}
rs4725102	1.71×10^{-1}	3.47×10^{-2}	8.36×10^{-7}	-7.58×10^{-3}	1.21×10^{-2}	5.32×10^{-1}	-7.99×10^{-2}	1.14×10^{-1}	4.85×10^{-1}

SNPs	GERD			Smoking Initiation			IPF		
	**Beta**	**SE**	***P*-value**	**Beta**	**SE**	***P*-value**	**Beta**	**SE**	***P*-value**
rs4730345	2.44×10^{-2}	9.24×10^{-3}	8.41×10^{-3}	1.83×10^{-2}	3.59×10^{-3}	3.38×10^{-7}	8.61×10^{-3}	3.19×10^{-2}	7.87×10^{-1}
rs4731959	-1.47×10^{-2}	1.27×10^{-2}	2.46×10^{-1}	-2.20×10^{-2}	4.47×10^{-3}	8.72×10^{-7}	-9.94×10^{-3}	4.41×10^{-2}	8.22×10^{-1}
rs4752018	2.65×10^{-2}	1.16×10^{-2}	2.23×10^{-2}	2.08×10^{-2}	4.23×10^{-3}	9.34×10^{-7}	7.61×10^{-2}	4.04×10^{-2}	5.93×10^{-2}
rs4811626	2.48×10^{-2}	9.25×10^{-3}	7.38×10^{-3}	-1.91×10^{-2}	3.60×10^{-3}	1.14×10^{-7}	1.63×10^{-2}	3.19×10^{-2}	6.10×10^{-1}
rs4911252	5.99×10^{-3}	9.23×10^{-3}	5.16×10^{-1}	1.90×10^{-2}	3.60×10^{-3}	1.32×10^{-7}	5.29×10^{-2}	3.19×10^{-2}	9.74×10^{-2}
rs508544	-1.65×10^{-2}	9.29×10^{-3}	7.49×10^{-2}	1.86×10^{-2}	3.56×10^{-3}	1.88×10^{-7}	-6.02×10^{-3}	3.21×10^{-2}	8.51×10^{-1}
rs530647	-4.59×10^{-4}	9.26×10^{-3}	9.60×10^{-1}	1.75×10^{-2}	3.57×10^{-3}	9.51×10^{-7}	-9.42×10^{-3}	3.21×10^{-2}	7.69×10^{-1}
rs55968130	3.70×10^{-3}	1.34×10^{-2}	7.83×10^{-1}	3.00×10^{-2}	5.90×10^{-3}	3.88×10^{-7}	4.16×10^{-2}	4.66×10^{-2}	3.72×10^{-1}
rs55974445	-8.21×10^{-4}	1.00×10^{-2}	9.35×10^{-1}	-2.49×10^{-2}	3.90×10^{-3}	1.59×10^{-10}	-4.73×10^{-2}	3.48×10^{-2}	1.74×10^{-1}
rs597041	1.35×10^{-2}	9.22×10^{-3}	1.42×10^{-1}	-1.78×10^{-2}	3.56×10^{-3}	6.17×10^{-7}	-7.13×10^{-2}	3.19×10^{-2}	8.23×10^{-1}
rs5995826	8.87×10^{-3}	9.41×10^{-3}	3.46×10^{-1}	1.84×10^{-2}	3.63×10^{-3}	4.27×10^{-7}	4.29×10^{-2}	3.26×10^{-2}	1.88×10^{-1}
rs6087600	-4.24×10^{-3}	9.26×10^{-3}	6.47×10^{-1}	-1.85×10^{-2}	3.76×10^{-3}	8.47×10^{-7}	-1.73×10^{-2}	3.21×10^{-2}	5.90×10^{-1}
rs61246633	-1.16×10^{-2}	1.95×10^{-3}	5.53×10^{-1}	-3.11×10^{-2}	6.07×10^{-3}	3.05×10^{-7}	1.02×10^{-2}	6.92×10^{-2}	8.83×10^{-1}
rs61805526	-1.99×10^{-2}	1.23×10^{-2}	1.05×10^{-1}	-2.73×10^{-2}	5.52×10^{-3}	7.74×10^{-7}	5.31×10^{-2}	4.21×10^{-2}	2.07×10^{-1}
rs62250723	1.17×10^{-2}	1.14×10^{-2}	3.04×10^{-1}	-2.13×10^{-2}	4.28×10^{-3}	6.75×10^{-7}	4.13×10^{-3}	3.96×10^{-2}	9.17×10^{-1}
rs6497753	-6.39×10^{-3}	1.02×10^{-2}	5.32×10^{-1}	1.89×10^{-2}	3.85×10^{-3}	8.87×10^{-7}	1.71×10^{-2}	3.53×10^{-2}	6.29×10^{-1}
rs6549362	1.21×10^{-2}	1.10×10^{-2}	2.69×10^{-1}	-1.93×10^{-2}	3.93×10^{-3}	9.42×10^{-7}	6.57×10^{-2}	3.80×10^{-2}	8.43×10^{-2}
rs6600376	-2.31×10^{-2}	1.00×10^{-2}	2.12×10^{-2}	1.88×10^{-2}	3.83×10^{-3}	9.73×10^{-7}	1.13×10^{-2}	3.47×10^{-2}	7.44×10^{-1}
rs6665145	-1.08×10^{-2}	1.00×10^{-2}	2.79×10^{-1}	-1.86×10^{-2}	3.69×10^{-3}	4.76×10^{-7}	-2.86×10^{-2}	3.48×10^{-2}	4.11×10^{-1}
rs6668412	-1.83×10^{-2}	9.27×10^{-3}	4.87×10^{-2}	1.83×10^{-2}	3.56×10^{-3}	2.63×10^{-7}	-1.14×10^{-2}	3.21×10^{-2}	7.22×10^{-1}
rs6691053	-8.99×10^{-3}	1.19×10^{-2}	4.49×10^{-1}	-2.20×10^{-2}	4.40×10^{-3}	5.38×10^{-7}	2.25×10^{-2}	4.10×10^{-2}	5.83×10^{-1}
rs66994942	3.68×10^{-3}	9.28×10^{-3}	6.92×10^{-1}	1.87×10^{-2}	3.70×10^{-3}	4.20×10^{-7}	8.61×10^{-3}	3.19×10^{-2}	7.87×10^{-1}

SNPs	GERD			Smoking Initiation			IPF		
	**Beta**	**SE**	***P*-value**	**Beta**	**SE**	***P*-value**	**Beta**	**SE**	***P*-value**
rs6717424	-2.41×10^{-2}	9.67×10^{-3}	1.27×10^{-2}	2.74×10^{-2}	3.60×10^{-3}	2.63×10^{-14}	-3.52×10^{-2}	3.21×10^{-2}	2.73×10^{-1}
rs6752675	6.27×10^{-3}	9.24×10^{-3}	4.98×10^{-1}	-2.55×10^{-2}	3.56×10^{-3}	7.18×10^{-13}	-1.14×10^{-2}	3.36×10^{-2}	7.35×10^{-1}
rs6786418	6.14×10^{-3}	9.77×10^{-3}	5.30×10^{-1}	-1.91×10^{-2}	3.88×10^{-3}	8.23×10^{-7}	-3.26×10^{-2}	3.20×10^{-2}	3.08×10^{-1}
rs6845883	-2.13×10^{-2}	1.10×10^{-2}	5.42×10^{-2}	-2.45×10^{-2}	4.32×10^{-3}	1.46×10^{-8}	8.39×10^{-3}	3.37×10^{-2}	8.04×10^{-1}
rs6911986	1.09×10^{-2}	1.03×10^{-2}	2.87×10^{-1}	2.10×10^{-2}	4.04×10^{-3}	1.94×10^{-7}	-5.03×10^{-2}	3.83×10^{-2}	1.89×10^{-1}
rs6974377	4.96×10^{-3}	9.87×10^{-3}	6.15×10^{-1}	2.01×10^{-2}	3.87×10^{-3}	1.91×10^{-7}	9.23×10^{-2}	3.49×10^{-2}	8.12×10^{-3}
rs6976111	-1.01×10^{-2}	9.60×10^{-3}	2.92×10^{-1}	2.67×10^{-2}	4.05×10^{-3}	3.99×10^{-11}	-1.51×10^{-2}	3.41×10^{-2}	6.58×10^{-1}
rs698050	-3.16×10^{-3}	9.20×10^{-3}	7.31×10^{-1}	1.76×10^{-2}	3.57×10^{-3}	7.66×10^{-7}	4.31×10^{-2}	3.32×10^{-2}	1.94×10^{-1}
rs7102620	-7.71×10^{-3}	9.27×10^{-3}	4.06×10^{-1}	-2.00×10^{-2}	3.66×10^{-3}	4.98×10^{-8}	3.56×10^{-2}	3.18×10^{-2}	2.63×10^{-1}
rs715693	5.91×10^{-3}	9.92×10^{-3}	5.51×10^{-1}	2.00×10^{-2}	3.65×10^{-3}	4.44×10^{-8}	4.83×10^{-2}	3.21×10^{-2}	1.33×10^{-1}
rs71627577	-1.33×10^{-2}	1.23×10^{-2}	2.78×10^{-1}	-3.02×10^{-2}	5.64×10^{-3}	8.59×10^{-8}	7.37×10^{-3}	3.43×10^{-2}	8.30×10^{-1}
rs717915	-5.38×10^{-3}	9.42×10^{-3}	5.68×10^{-1}	1.92×10^{-2}	3.70×10^{-3}	2.05×10^{-7}	5.74×10^{-2}	4.22×10^{-2}	1.73×10^{-1}
rs72678859	-9.90×10^{-3}	1.33×10^{-2}	4.57×10^{-1}	-2.40×10^{-2}	4.83×10^{-3}	6.30×10^{-7}	7.65×10^{-2}	3.26×10^{-2}	1.89×10^{-2}
rs72683129	9.28×10^{-3}	1.34×10^{-2}	4.88×10^{-1}	-2.52×10^{-2}	5.06×10^{-3}	6.85×10^{-7}	-4.86×10^{-2}	4.60×10^{-2}	2.91×10^{-1}
rs72861745	2.67×10^{-2}	1.22×10^{-2}	2.88×10^{-2}	-2.90×10^{-2}	4.15×10^{-3}	2.90×10^{-12}	6.07×10^{-2}	4.61×10^{-2}	1.89×10^{-1}
rs72896886	-2.99×10^{-2}	1.24×10^{-2}	1.64×10^{-2}	-2.69×10^{-2}	4.84×10^{-3}	2.75×10^{-8}	-3.01×10^{-2}	4.27×10^{-2}	4.82×10^{-1}
rs72976162	3.65×10^{-3}	1.20×10^{-2}	7.62×10^{-1}	2.22×10^{-2}	4.53×10^{-3}	9.21×10^{-7}	4.62×10^{-3}	4.32×10^{-2}	9.15×10^{-1}
rs7333724	6.94×10^{-3}	1.20×10^{-2}	5.62×10^{-1}	-2.18×10^{-2}	4.34×10^{-3}	5.26×10^{-7}	-1.08×10^{-2}	4.19×10^{-2}	7.96×10^{-1}
rs74531549	-3.81×10^{-2}	1.65×10^{-2}	2.12×10^{-2}	2.69×10^{-2}	5.19×10^{-3}	2.11×10^{-7}	-3.35×10^{-2}	4.16×10^{-2}	4.20×10^{-1}
rs745571	-4.07×10^{-3}	9.19×10^{-3}	6.58×10^{-1}	-1.77×10^{-2}	3.56×10^{-3}	6.55×10^{-7}	-5.90×10^{-2}	5.73×10^{-2}	3.03×10^{-1}
rs74876138	4.03×10^{-3}	9.64×10^{-3}	6.76×10^{-1}	2.00×10^{-2}	4.05×10^{-3}	7.42×10^{-7}	-8.64×10^{-3}	3.18×10^{-2}	7.86×10^{-1}
rs7505855	-1.60×10^{-2}	9.30×10^{-3}	8.48×10^{-2}	-1.90×10^{-2}	3.61×10^{-3}	1.45×10^{-7}	1.67×10^{-2}	3.32×10^{-2}	6.16×10^{-1}

SNPs	GERD			Smoking Initiation			IPF		
	**Beta**	**SE**	***P*-value**	**Beta**	**SE**	***P*-value**	**Beta**	**SE**	***P*-value**
rs75121303	1.26×10^{-3}	1.30×10^{-2}	9.22×10^{-1}	-2.67×10^{-2}	4.91×10^{-3}	5.25×10^{-8}	-8.15×10^{-2}	4.48×10^{-2}	6.88×10^{-2}
rs75177132	-4.93×10^{-3}	1.95×10^{-2}	8.00×10^{-1}	-4.65×10^{-2}	8.38×10^{-3}	2.85×10^{-8}	-6.57×10^{-2}	6.67×10^{-2}	3.25×10^{-1}
rs7545279	1.43×10^{-3}	1.01×10^{-2}	8.88×10^{-1}	-2.31×10^{-2}	4.35×10^{-3}	1.15×10^{-7}	4.40×10^{-2}	3.49×10^{-2}	2.08×10^{-1}
rs7594223	-9.19×10^{-3}	9.72×10^{-3}	3.45×10^{-1}	-1.88×10^{-2}	3.66×10^{-3}	2.89×10^{-7}	3.93×10^{-2}	3.37×10^{-2}	2.43×10^{-1}
rs7631379	3.73×10^{-3}	1.26×10^{-2}	7.68×10^{-1}	2.19×10^{-2}	4.40×10^{-3}	6.58×10^{-7}	5.49×10^{-2}	4.41×10^{-2}	2.13×10^{-1}
rs766614	4.22×10^{-4}	1.04×10^{-2}	9.68×10^{-1}	2.28×10^{-2}	4.34×10^{-3}	1.51×10^{-7}	3.67×10^{-2}	3.59×10^{-2}	3.07×10^{-1}
rs7707036	2.51×10^{-3}	9.21×10^{-3}	7.85×10^{-1}	1.77×10^{-2}	3.57×10^{-3}	7.24×10^{-7}	-3.70×10^{-2}	3.18×10^{-2}	2.45×10^{-1}
rs77075313	-1.62×10^{-2}	2.08×10^{-2}	4.37×10^{-1}	-4.29×10^{-2}	8.70×10^{-3}	8.15×10^{-7}	2.16×10^{-2}	7.22×10^{-2}	7.65×10^{-1}
rs773107	-2.37×10^{-2}	1.03×10^{-2}	2.09×10^{-2}	-1.94×10^{-2}	3.97×10^{-3}	9.58×10^{-7}	-5.92×10^{-2}	3.55×10^{-2}	9.54×10^{-2}
rs77963899	1.72×10^{-2}	2.20×10^{-2}	4.35×10^{-1}	-4.02×10^{-2}	7.71×10^{-3}	1.93×10^{-7}	3.49×10^{-3}	7.56×10^{-2}	9.63×10^{-1}
rs7835456	3.28×10^{-3}	9.18×10^{-3}	7.21×10^{-1}	-2.00×10^{-2}	3.56×10^{-3}	1.96×10^{-8}	-5.75×10^{-2}	3.18×10^{-2}	7.05×10^{-2}
rs7844990	-8.25×10^{-3}	9.30×10^{-3}	3.75×10^{-1}	2.01×10^{-2}	3.63×10^{-3}	3.23×10^{-8}	-1.67×10^{-2}	3.22×10^{-2}	6.05×10^{-1}
rs7855587	9.79×10^{-3}	9.43×10^{-3}	2.99×10^{-1}	1.93×10^{-2}	3.56×10^{-3}	5.57×10^{-8}	8.49×10^{-2}	3.23×10^{-2}	8.60×10^{-3}
rs78975669	-3.05×10^{-2}	1.75×10^{-2}	8.25×10^{-2}	-4.28×10^{-2}	8.67×10^{-3}	7.86×10^{-7}	6.42×10^{-3}	6.00×10^{-2}	9.15×10^{-1}
rs7901883	1.36×10^{-2}	1.09×10^{-2}	2.13×10^{-1}	-2.45×10^{-2}	4.25×10^{-3}	7.98×10^{-9}	-8.78×10^{-2}	3.77×10^{-2}	1.97×10^{-2}
rs79098346	-2.87×10^{-2}	1.65×10^{-2}	8.21×10^{-2}	3.39×10^{-2}	6.87×10^{-3}	7.84×10^{-7}	-5.57×10^{-2}	5.69×10^{-2}	3.28×10^{-1}
rs7946110	5.92×10^{-2}	1.19×10^{-2}	5.92×10^{-7}	4.87×10^{-4}	4.85×10^{-3}	9.20×10^{-1}	-5.83×10^{-2}	4.05×10^{-2}	1.50×10^{-1}
rs7996220	8.20×10^{-3}	9.21×10^{-3}	3.73×10^{-1}	1.83×10^{-2}	3.57×10^{-3}	2.85×10^{-7}	7.49×10^{-4}	3.19×10^{-2}	9.81×10^{-1}
rs8010469	-4.36×10^{-4}	9.21×10^{-3}	9.62×10^{-1}	-1.77×10^{-2}	3.57×10^{-3}	7.56×10^{-7}	3.85×10^{-2}	3.19×10^{-2}	2.28×10^{-1}
rs8082894	1.02×10^{-2}	1.00×10^{-2}	3.08×10^{-1}	-1.89×10^{-2}	3.83×10^{-3}	8.53×10^{-7}	-8.93×10^{-2}	3.48×10^{-2}	1.02×10^{-2}
rs814518	3.86×10^{-3}	9.51×10^{-3}	6.85×10^{-1}	-1.90×10^{-2}	3.84×10^{-3}	7.71×10^{-7}	-1.44×10^{-2}	3.28×10^{-2}	6.61×10^{-1}
rs827153	-1.30×10^{-3}	9.71×10^{-3}	8.94×10^{-1}	1.80×10^{-2}	3.68×10^{-3}	9.49×10^{-7}	-3.51×10^{-2}	3.37×10^{-2}	2.97×10^{-1}

SNPs	GERD			Smoking Initiation			IPF		
	**Beta**	**SE**	***P*-value**	**Beta**	**SE**	***P*-value**	**Beta**	**SE**	***P*-value**
rs914647	1.77×10^{-3}	9.35×10^{-3}	8.50×10^{-1}	1.79×10^{-2}	3.61×10^{-3}	7.60×10^{-7}	4.07×10^{-3}	3.23×10^{-2}	9.00×10^{-1}
rs917154	-1.43×10^{-4}	1.10×10^{-2}	9.90×10^{-1}	-2.91×10^{-2}	4.90×10^{-3}	2.77×10^{-9}	4.10×10^{-2}	3.80×10^{-2}	2.81×10^{-1}
rs929623	-2.05×10^{-2}	1.04×10^{-2}	4.83×10^{-2}	-1.90×10^{-2}	3.76×10^{-3}	4.60×10^{-7}	-1.20×10^{-2}	3.61×10^{-2}	7.38×10^{-1}
rs9388686	1.28×10^{-3}	1.16×10^{-2}	9.12×10^{-1}	2.35×10^{-2}	4.70×10^{-3}	6.14×10^{-7}	-1.42×10^{-2}	4.02×10^{-2}	7.25×10^{-1}
rs9399695	2.64×10^{-3}	9.37×10^{-3}	7.78×10^{-1}	-1.86×10^{-2}	3.59×10^{-3}	2.37×10^{-7}	-2.27×10^{-2}	3.24×10^{-2}	4.84×10^{-1}
rs9411424	-1.40×10^{-2}	1.02×10^{-2}	1.71×10^{-1}	-1.89×10^{-2}	3.82×10^{-3}	6.95×10^{-7}	-7.68×10^{-3}	3.56×10^{-2}	8.29×10^{-1}
rs9423279	6.43×10^{-3}	9.42×10^{-3}	4.95×10^{-1}	-2.05×10^{-2}	3.71×10^{-3}	3.21×10^{-8}	3.45×10^{-2}	3.26×10^{-2}	2.90×10^{-1}
rs943725	-8.58×10^{-3}	9.77×10^{-3}	3.80×10^{-1}	-1.93×10^{-2}	3.94×10^{-3}	9.96×10^{-7}	3.71×10^{-2}	3.39×10^{-2}	2.74×10^{-1}
rs9518075	-2.01×10^{-2}	1.10×10^{-2}	6.79×10^{-2}	-2.19×10^{-2}	4.28×10^{-3}	3.22×10^{-7}	-6.04×10^{-2}	3.80×10^{-2}	1.12×10^{-1}
rs954467	-1.20×10^{-2}	9.39×10^{-3}	2.00×10^{-1}	1.84×10^{-2}	3.56×10^{-3}	2.20×10^{-7}	-3.51×10^{-2}	3.25×10^{-2}	2.81×10^{-1}
rs9545502	-2.00×10^{-2}	1.06×10^{-2}	5.95×10^{-2}	-2.11×10^{-2}	4.28×10^{-3}	8.23×10^{-7}	-3.79×10^{-2}	3.68×10^{-2}	3.03×10^{-1}
rs9582795	-9.66×10^{-3}	1.10×10^{-2}	3.78×10^{-1}	-1.90×10^{-2}	3.80×10^{-3}	5.78×10^{-7}	-4.41×10^{-2}	3.81×10^{-2}	2.47×10^{-1}
rs9847024	3.23×10^{-3}	1.05×10^{-2}	7.58×10^{-1}	2.21×10^{-2}	4.19×10^{-3}	1.28×10^{-7}	-1.22×10^{-2}	3.65×10^{-2}	7.37×10^{-1}
rs9881798	7.14×10^{-3}	9.47×10^{-3}	4.51×10^{-1}	1.83×10^{-2}	3.62×10^{-3}	4.05×10^{-7}	3.31×10^{-3}	3.28×10^{-2}	9.20×10^{-1}
rs9933873	-6.01×10^{-4}	9.23×10^{-3}	9.48×10^{-1}	1.77×10^{-2}	3.58×10^{-3}	7.09×10^{-7}	-2.30×10^{-2}	3.20×10^{-2}	4.73×10^{-1}
rs9936337	3.45×10^{-2}	9.63×10^{-3}	3.41×10^{-4}	1.91×10^{-2}	3.58×10^{-3}	1.05×10^{-7}	3.62×10^{-2}	3.33×10^{-2}	2.77×10^{-1}

Abbreviations: SNP: single nucleotide polymorphism; SE: standard error; GERD: gastroesophageal reflux disease; IPF: idiopathic pulmonary fibrosis.

Table S6 Results of MVMR adjusting for smoking initiation

Method	Cohort	OR (95% CI)	P-value
**GRED on susceptibility of IPF**		**OR (95% CI)**	
IVW	Discovery	1.30 (0.93 – 1.68)	0.071
MVMR -Egger		0.91 (0.34 – 1.48)	0.767
**GRED on susceptibility of IPF**		**OR (95% CI)**	
IVW	Replication	1.14 (0.74 – 1.54)	0.464
MVMR-Egger		1.07 (0.55 – 1.59)	0.788

Abbreviations: MVMR: multivariate Mendelian randomization; GERD: gastroesophageal reflux disease; IPF: idiopathic pulmonary fibrosis; OR: odds ratio; CI: confidence intervals; IVW: inverse variance weighted.

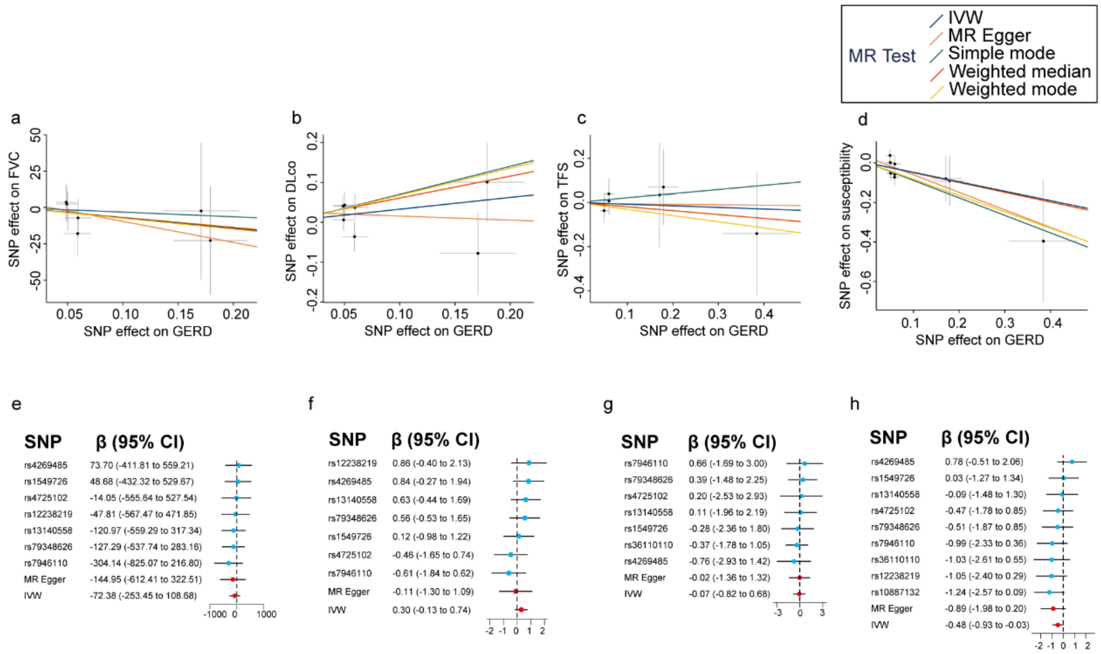

Fig. S1 Scatterplots and forest plots of associations between GERD-associated SNPs and IPF in replication cohort. Scatterplots of SNP effects on GERD and FVC (a), DLco (b), TFS (c), and susceptibility (d) in patients with IPF. Forest plots of individual and combined SNP MR-estimated effect size for GERD on FVC (e), DLco (f), TFS (g), and susceptibility (h) in patients with IPF. Abbreviations: MR: Mendelian randomization; IVW: inverse variance weighted; GERD: gastroesophageal reflux disease; FVC: forced vital capacity; DLco: diffuse lung capacity for carbon monoxide; TFS: transplantation-free survival; SNP: single nucleotide polymorphism.

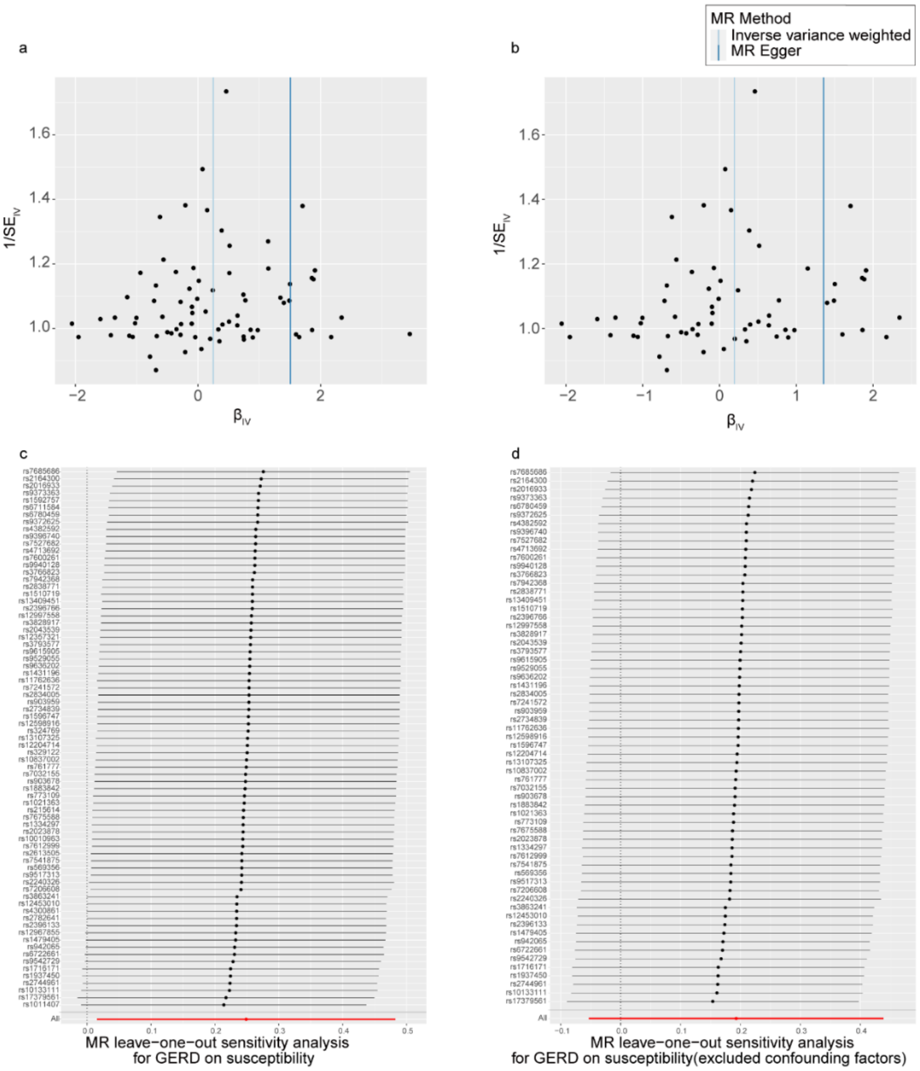

Fig. S2 Funnel plots and leave-one-out analyses for SNPs associated with GERD on susceptibility to IPF in discovery cohort. Funnel plots showed the overall heterogeneity effect of GERD on susceptibility to IPF before removing SNPs related to confounding factors and outliers (a), and after removal (b). The leave-one-out analyses were conducted to assess the influence of individual SNP variants of GERD susceptibility to IPF before removing SNPs related to confounding factors and outliers (a), and after removal (b). Abbreviations: MR: Mendelian randomization; GERD: gastroesophageal reflux disease; IPF: idiopathic pulmonary fibrosis; SNP: single nucleotide polymorphism; SE: standard error; IV: instrumental variable.

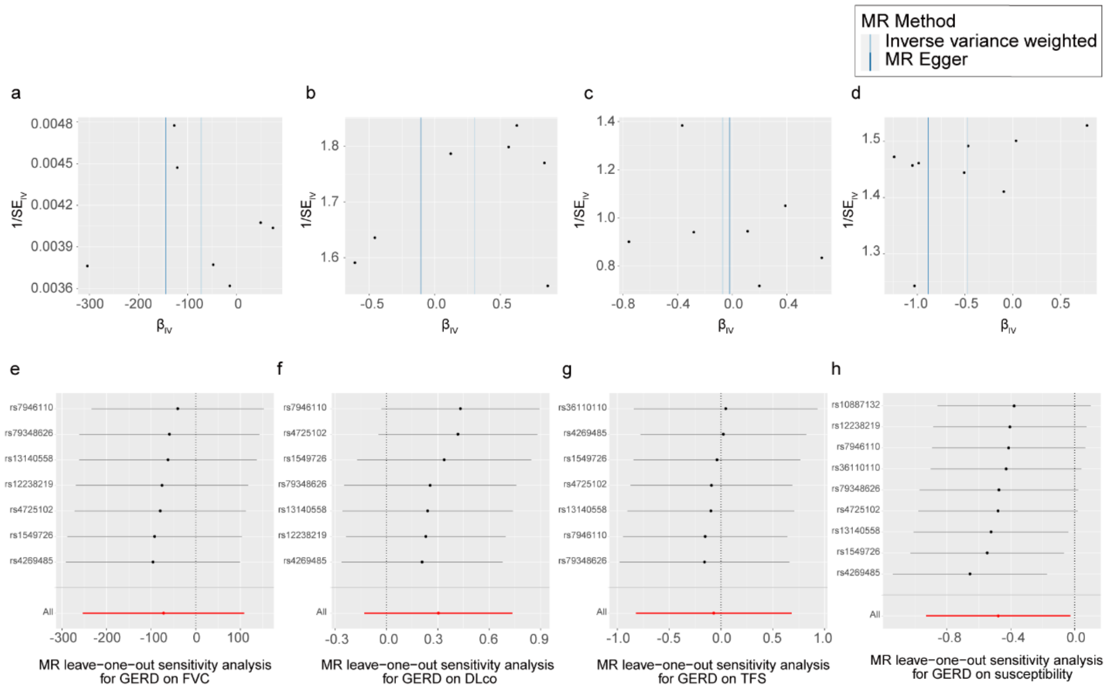

Fig. S3 Funnel plots and leave-one-out analyses for SNPs associated with GERD on IPF in replication cohort. Funnel plots showed the overall heterogeneity effect of GERD on FVC (a), DLco (b), TFS (c), and susceptibility (d) in patients with IPF. The leave-one-out analyses were conducted to assess the influence of individual SNP variants of GERD on FVC (e), DLco (f), TFS (g), and susceptibility (h) in patients with IPF. Abbreviations: MR: Mendelian randomization; GERD: gastroesophageal reflux disease; FVC: forced vital capacity; DLco: diffuse lung capacity for carbon monoxide; TFS: transplantation-free survival; SNP: single nucleotide polymorphism; SE: standard error; IV: instrumental variable; IPF: idiopathic pulmonary fibrosis.

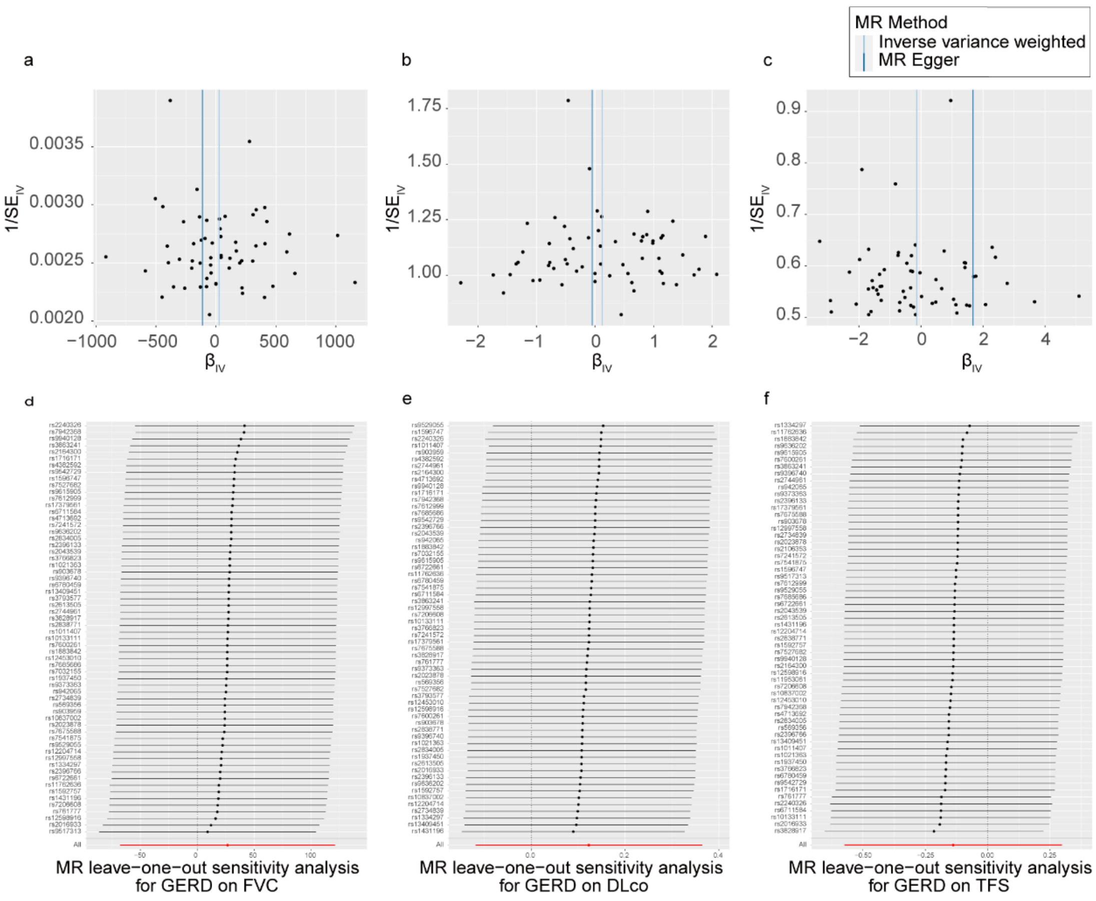

Fig. S4 Funnel plots and leave-one-out analyses for SNPs associated with GERD on the prognosis of IPF in discovery cohort. Funnel plots showed the overall heterogeneity effect of GERD on FVC (a), DLco (b), and TFS (c) in patients with IPF. The leave-one-out analyses were conducted to assess the influence of individual SNP variants of GERD on FVC (d), DLco (e), and TFS (f). Abbreviations: MR: Mendelian randomization; GERD: gastroesophageal reflux disease; FVC: forced vital capacity; DLco: diffuse lung capacity for carbon monoxide; TFS: transplantation-free survival; SNP: single nucleotide polymorphism; SE: standard error; IV: instrumental variable; IPF: idiopathic pulmonary fibrosis.
